# A single-stranded based library preparation method for virome characterization

**DOI:** 10.1186/s40168-024-01935-5

**Published:** 2024-10-24

**Authors:** Xichuan Zhai, Alex Gobbi, Witold Kot, Lukasz Krych, Dennis Sandris Nielsen, Ling Deng

**Affiliations:** 1 Section for Food Microbiology, Gut Health and Fermentation, Department of Food Science, University of Copenhagen, Rolighedsvej 26, Frederiksberg C, 1958 Denmark; 2 Section of Microbial Ecology and Biotechnology, Department of Plant and Environmental Sciences, University of Copenhagen, Thorvaldsensvej 40, 1871 Frederiksberg C, Denmark; 3 Department of Agricultural, Forestry, Food Sciences (DISAFA), University of Turin, Largo P. Braccini, 2, Grugliasco, Torino, 10095 Italy

**Keywords:** Single-stranded library, Phage mock community, SsDNA virome, DsDNA virome, RNA virome, Gut virome, Modified nucleotides

## Abstract

**Background:**

The gut virome is an integral component of the gut microbiome, playing a crucial role in maintaining gut health. However, accurately depicting the entire gut virome is challenging due to the inherent diversity of genome types (dsDNA, ssDNA, dsRNA, and ssRNA) and topologies (linear, circular, or fragments), with subsequently biases associated with current sequencing library preparation methods. To overcome these problems and improve reproducibility and comparability across studies, universal or standardized virome sequencing library construction methods are highly needed in the gut virome study.

**Results:**

We repurposed the ligation-based single-stranded library (SSLR) preparation method for virome studies. We demonstrate that the SSLR method exhibits exceptional efficiency in quantifying viral DNA genomes (both dsDNA and ssDNA) and outperforms existing double-stranded (Nextera) and single-stranded (xGen, MDA + Nextera) library preparation approaches in terms of minimal amplification bias, evenness of coverage, and integrity of assembling viral genomes. The SSLR method can be utilized for the simultaneous library preparation of both DNA and RNA viral genomes. Furthermore, the SSLR method showed its ability to capture highly modified phage genomes, which were often lost using other library preparation approaches.

**Conclusion:**

We introduce and improve a fast, simple, and efficient ligation-based single-stranded DNA library preparation for gut virome study. This method is compatible with Illumina sequencing platforms and only requires ligation reagents within 3-h library preparation, which is similar or even better than the advanced library preparation method (xGen). We hope this method can be further optimized, validated, and widely used to make gut virome study more comparable and reproducible.

Video Abstract

**Supplementary Information:**

The online version contains supplementary material available at 10.1186/s40168-024-01935-5.

## Introduction

The gut microbiome constitutes a diverse array of microbes, comprising bacteria, archaea, viruses/bacteriophages, and fungi, residing in the human intestine [1]. Bacteriophages (or phages for short), among them, are believed to be highly abundant constituents of the human microbiome possibly equal in numbers to bacteria. They play important roles in modulating the diversity and abundance of gut bacteria, maintaining a dynamic equilibrium [1, 2]. There is growing evidence linking gut virome dysbiosis to human diseases such as inflammatory bowel disease [3–5], severe acute malnutrition [6], alcoholic liver disease [7], type 2 diabetes [8], stunting [9], and recently also asthma [10]. Studies have also demonstrated that in fecal microbiota transplantation to treat, e.g., recurrent *Clostridioides difficile* (*rCdiff*), the virome component is important for treatment efficacy, and even that sterile-filtered feces used for the so-called fecal filtrate transplantation is able to cure *rCdiff* [11–13]. Consequently, there is growing interest in studying the gut virome to understand its role in various diseases and its potential clinical applications.

However, this effort is hampered by persistent biases and inconsistencies across studies due to non-standardized and non-optimized pipelines. Every stage of the virome exploration process, from sample collection to bioinformatic analysis, is crucial, with virome characterization of complex microbial communities relying on essential steps such as virus-like particle isolation/purification, viral DNA/RNA purification, and sequencing library preparation and shotgun high-throughput sequencing [14–17]. Furthermore, despite advances during recent years, virus identification is still challenging due to their variability, small genome sizes, and genetic mosaicism. Further, virome studies (distinct from bacteriome studies) present a unique challenge with diverse genome types (dsDNA, ssDNA, dsRNA, and ssRNA) and different topologies (linear, circular, or segments) [18, 19], complicating library preparation and down-stream analysis [1, 3, 20] and emphasizing the need for universal or standardized virome sequencing library construction methods for improved reproducibility and comparability across studies.

Commonly used library preparation methods, such as randomly amplified and linker-amplified shotgun libraries, have limitations, as these methods are restricted to target only dsDNA viruses and can result in uneven coverage of viral genomes, particularly when the viruses are very different in proportional abundances [19, 21]. The transposon-based method, while fast and requiring low-input template, is still restricted to dsDNA [22]. To overcome these limitations, multiple displacement amplification (MDA) has been utilized, increasing DNA amounts for low biomass virome samples and converting ssDNA into dsDNA for further library preparation. However, MDA distorts the ratios of different viral DNA forms by over-amplifying small circular ssDNA genomes and unevenly amplifying linear genomes [23–25]. More recently, single-stranded-based methods for library construction have been applied, such as the xGen ssDNA and Low-Input DNA Library Preparation Kit from IDT (previous name Accel-NGS kit from Swift Biosciences). Though promising, the cost per sample is relatively high, and it involves multiple purification steps lowering throughput.

Currently, RNA viruses, especially dsRNA viruses, are significantly undersampled due to the instability of RNA and its incompatibility with common DNA sequencing library preparations [26, 27]. As a result, DNA and RNA viruses are analyzed separately [28]. While recent studies have revealed a greater diversity of environmental RNA viruses than previously thought [26, 29, 30], little information is available on human gut RNA viral communities [31]. The commonly used RNA-Seq technology for investigating RNA viruses requires large amounts of sample material, and the preparation process is expensive and time-consuming and can be further complicated by DNA contamination [32]. To overcome these challenges, innovative methods like the Novel enrichment technique of VIRomes (NetoVIR) and Full-Length Double-Stranded RNA Sequencing (FLDS) have been developed. However, these techniques introduce their own biases, particularly during library preparation. NetoVIR, despite providing a modular, customizable, and reproducible approach, introduces biases from whole transcriptome amplification (WTA) and multiple displacement amplification (MDA), which can affect the accuracy of the virome composition [33]. Similarly, FLDS, which is designed to comprehensively detect intracellular dsRNA, inherently limits its application for virome studies and faces additional hurdles due to the complexity of its library preparation process [34].

To address these challenges, we drew inspiration from the Single Reaction Single-stranded LibrarY (SRSLY) method, originally designed for the library preparation of cell-free DNA and oligo sequencing [35]. We have extended and improved this method for gut virome sequencing (both DNA and RNA) with cost-effectiveness and time-saving in mind. We compared the capability and quantitative accuracy of SRSLY with three different virome library preparation methods (Nextera, MDA amplification, and xGen) for Illumina short-read sequencing. We evaluated the types and levels of sequencing bias generated by these protocols using a wide range of DNA/RNA phage mock communities and performed qualitative and quantitative analyses using a diverse mock community with different ratios of four different genome types (dsDNA, ssDNA, dsRNA, and ssRNA) to further verify the method’s applicability for virome studies. Additionally, we assessed reliability, including error rates, composition bias, and assembling accuracy of these methods. Finally, we validated the utility and performance of the methods for human fecal virome analysis.

## Results

### SSLR is an effective method for quantifying DNA phage genomes

**Fig. 1:**
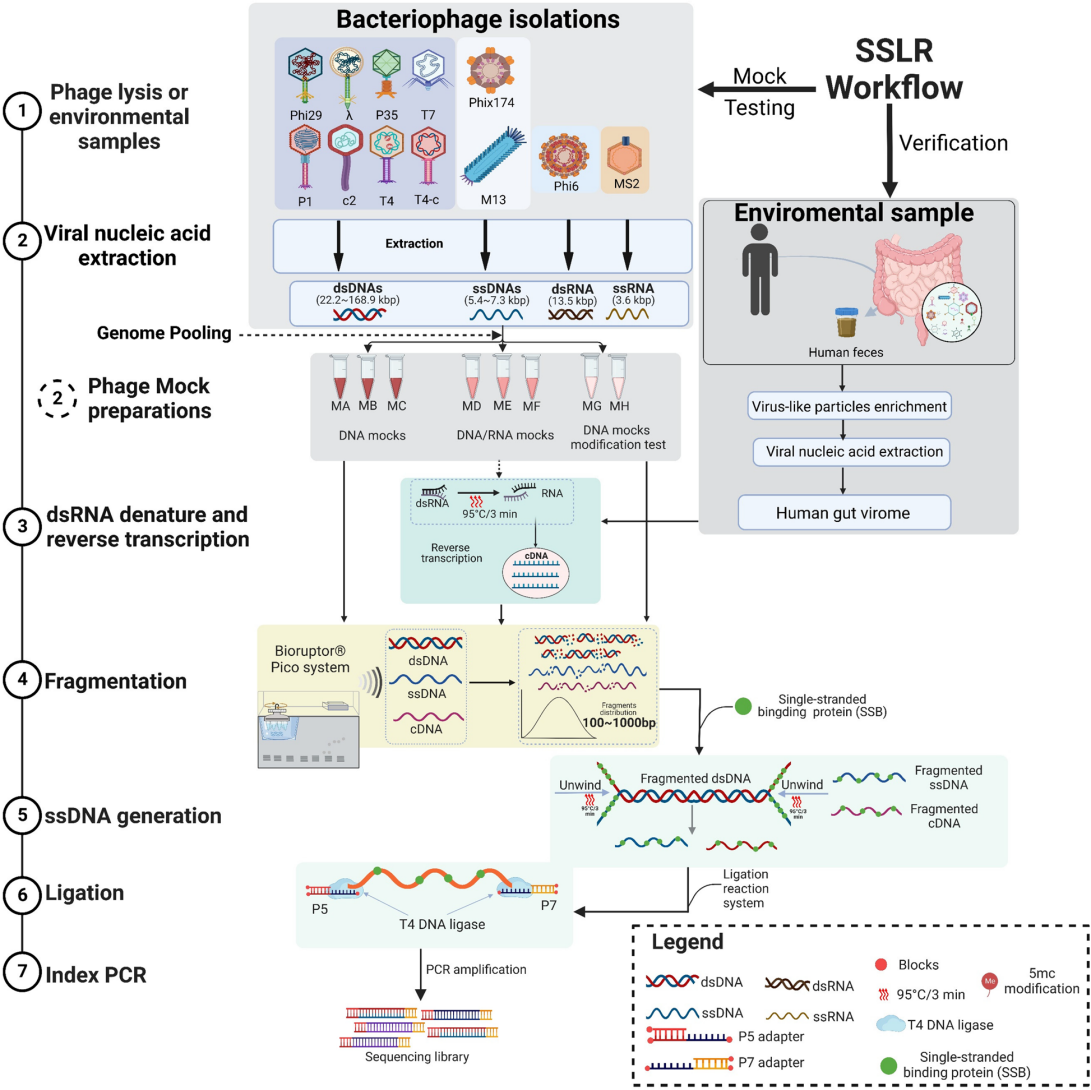
Overview of study workflow. The reproposed library preparation method (SSLR) was indicated with seven steps (detailed information can be found from Fig. S1 and Supplementary file 1). Five different libraries were used to prepare and sequence three artificial bacteriophage mocks containing different proportions of the ssDNA phages (phiX174 and M13mp18) mixed with the dsDNA phages. These phage genome abundance values were calculated based on the quantity of dsDNA and ssDNA phages measured Qubit dsDNA (or ssDNA) HS Assay kit. MA, Mock A with a ratio of ~ 90:10 for dsDNA and ssDNA (Fig. S1E); MB, Mock B with a ratio of ~ 50:50 for dsDNA and ssDNA; MC, Mock C with a ratio of ~ 10:90 for dsDNA and ssDNA. MD, Mock D with a ratio of ~ 90:10 for DNA and RNA; ME, Mock E with a ratio of ~ 50:50 for DNA and RNA; MF, Mock F with a ratio of 10:90 for DNA and RNA (Fig. S1F). MG, Mock G contains the highly modified dsDNA T4 genome (T4) with equal ratio of all genomes; MH, Mock H contains less modified dsDNA T4 genome (T4-c) with equal ratio of all genomes (Fig. S1G)

**Fig. 2:**
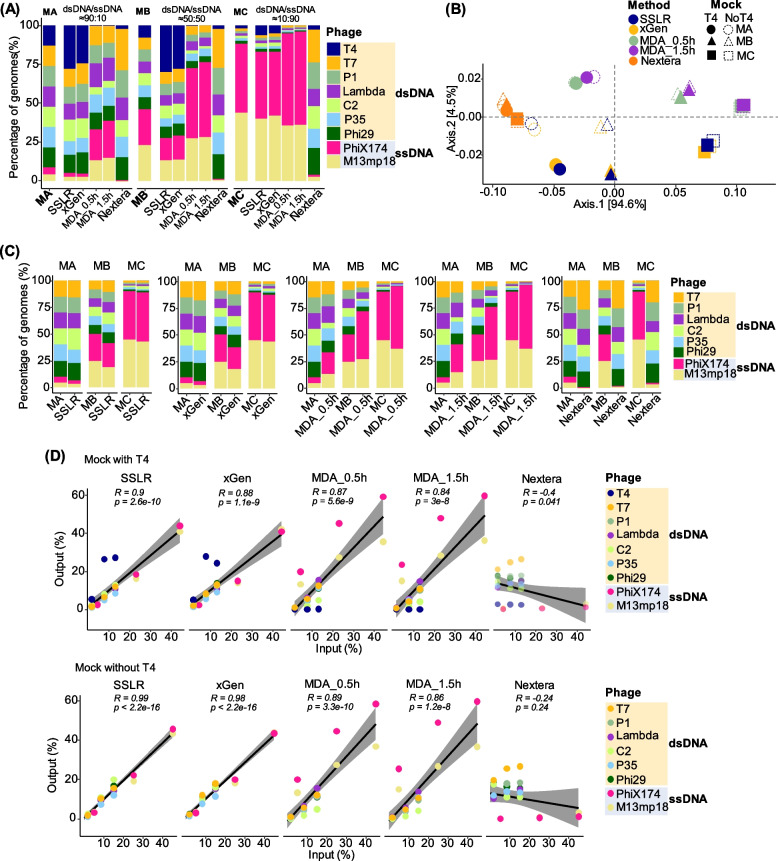
Comparison of different library strategies for DNA mock communities. **A** Percentage of phage genomes with dsDNA T4 generated by different library preparation methods. Different colors indicate different phage genomes. **B** Principal coordinates analysis (PCoA) plots of bray–Curtis distance matrices. PCoA was used to plot the beta diversity of mock-associated communities using the bray matrix. Different colors indicate different library preparation methods, and different shapes indicate different DNA mock communities. The dark-filled shapes display the mock with dsDNA T4 genome and the non-filled without dsDNA T4. For each axis, in square brackets, the percentage of variation explained was reported. **C** Percentage of phage genomes without dsDNA T4 genome. **D** Pearson correlation coefficient (r) and two-tailed *p*-value between the expected and obtained read distributions (in percentage) in different DNAs phage mock communities with (top panel) or without (bottom panel) phage T4 genome. Five different libraries were used to prepare and sequence three artificial bacteriophage mocks containing different proportions of the ssDNA phage (phiX174 and M13mp18) mixed with the dsDNA phage. These phage genome abundance values were calculated based on the quantity of dsDNA and ssDNA phages measured Qubit dsDNA (or ssDNA) HS Assay kit. MA, Mock A with a ratio of ~ 90:10 for dsDNA and ssDNA; MB, Mock B with a ratio of ~ 50:50 for dsDNA and ssDNA; MC, Mock C with a ratio of ~ 10:90 for dsDNA and ssDNA

We conducted sequencing library preparation using three DNA mock communities (Mock A, B, and C; Fig. 1, Fig. S1A and E) containing different ratios of dsDNA to ssDNA phages (10%, 50%, and 90% of dsDNA, Table S1) with five different methods, namely Nextera, MDA_0.5 h, MDA_1.5 h (0.5 h and 1.5 h referring to the amplification time), xGen, and SSLR. Overall, all the phage genomes were detectable even when the total dsDNA inputs were as low as 0.20 ng in the high ssDNA genomes mock (Mock C, Fig. 2A). However, the efficiency of quantification varied between methods. Nextera library preparation significantly underestimated ssDNA phages (24- to 35-fold, Fig. 2A and Table S1), where only 7 of 9 genomes could be captured (Table S2), making it unsuitable for ssDNA genomes studies. Although MDA can be very helpful for ssDNA genome studies, we observed a selective amplification bias of ssDNAs (threefold, Table S1) even with short-time amplification (MDA_0.5 h). Notably, no significant amplification biases were observed in mocks with a high percentage of ssDNA genomes (≥ 50%, about onefold, Table S1). In contrast, both SSLR and xGen accurately recovered the percentage of ssDNA genomes when present in high ratios (≥ 50%, Mock B and C) but slightly underestimated ssDNA genomes when present in low ratio inputs (about 10%, Mock A, Fig. S2A).

Interestingly, we observed quite different quantifications of the dsDNA T4 genome prepared with different methods (Fig. 2A, B, D and Table S1). Less than 2% of dsDNA T4 genomes were sequenced in all the MDA-related libraries, while 23 to 34% were reached in the Nextera libraries compared to their corresponding inputs. In contrast, both SSLR and xGen overestimated T4 genomes (1.8- to 3.6-folds, we later found this is due to under-quantification of dsDNA T4 DNA concentration using Qubit for mock construction, see below), especially in the mock with low dsDNA T4 genome input (Mock C, Fig. 2A, D, and Table S1).

We further examined the quantification of viral genomes without considering dsDNA T4 genome in the mocks (Fig. 2C and D) and found that the percentage of each genome was consistently accurate across different mock communities when the libraries were constructed by SSLR or xGen. These methods showed strong linear correlations between expected and observed percentage distributions (*R* = 0.99, *p* < 2.2e-16, Fig. 2D). Genome size and GC content of each genome had minimal influence on these quantifications (Fig. S2B and C), and both methods provided a similar overview of DNA phage communities (Fig. 2B). While the variations could be minimized for the MDA-related methods without including the T4 genome, the quantifications of ssDNA (especially phiX174) were less consistent than the quantification of dsDNA genomes due to their much higher sequencing depths (Fig. S3 and Table S1). The quantification of phages using the Nextera method appeared to be more consistent when the dsDNA phages were calculated exclusively (Fig. 2B and C).

## SSLR has less *bias* and higher accuracy in sequencing DNA phages compared to existing methods

We then assessed the efficiency and accuracy of the library prepared using different methods with the presence of the dsDNA T4 genome. The overall mapping rate to the mock genomes from SSLR method was comparable to xGen but higher than Nextera and MDA-related libraries (Fig. S4A). Furthermore, SSLR had a lower error rate for the entire mock community compared to Nextera and MDA methods, similar to the rate produced by xGen method (Fig. S4B and C).

Upon analyzing each genomes prepared from SSLR and xGen, we observed mostly uniform coverage across the genomes, except for sharp spikes near the start and end of each genome (Fig.S3). However, the coverage of ssDNA genomes was significantly increased in MDA-related methods and largely neglected by the Nextera method (Fig. S3 and Fig. S4D). Additionally, the effect of GC content on individual genome profiles (GC bias) varied among different library preparation methods. MDA-related and Nextera libraries showed a similar pattern with coverage increasing as GC content increased. However, coverage biases from SSLR and xGen were not apparently associated with their GC content (Fig. S5).

Finally, we evaluated the assembly accuracy and efficiency of the DNA phage genomes in the mock community prepared with different library methods. At a same sequencing depth, the assembly from the SSLR prepared library exhibited high quality, including the fully reconstructed dsDNA phage T4 genome (Table S3). In contrast, the other library methods provided less integrity with large gaps in the reconstructed genomes. Moreover, the hits of assembled contigs to the reference genomes from the SSLR prepared library showed one contig per reference genome (except for phage P1, where SSLR led to 2 contigs for dsDNA P1 genome, Table S4), where the other methods resulted wide range hits from 0 to 22.

## SSLR can quantify highly modified viral DNA

**Fig. 3:**
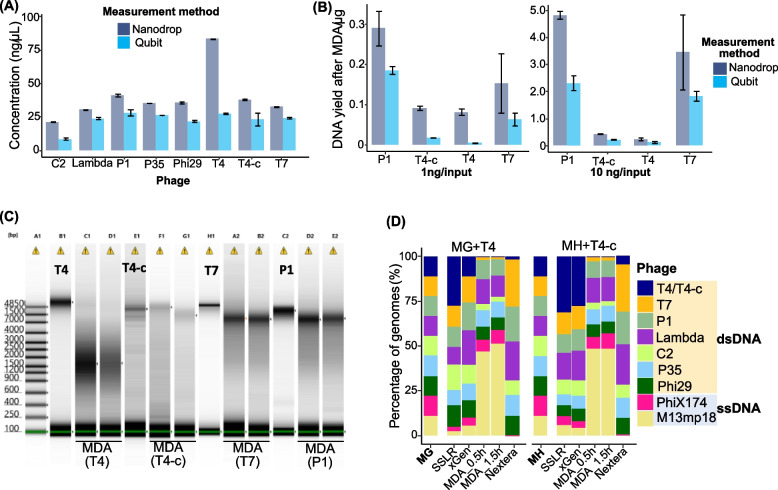
Quantification and MDA bias for highly modified T4 genome. **A** The concentrations of DNAs used in this study were measured by two different quantification methods (Qubit and NanoDrop). **B** MDA amplification differs in genomes. One and 10 ng of DNA from dsDNA P1, T4, T4-c, and T7 were amplified by 30 min of MDA and purified with Zymol Genomic Purification kit. The *y*-axis indicates the total yield of amplified MDA products (μg) and measured either by NanoDrop (red) or Qubit (blue). **C** The MDA amplification dsDNA products (T4, T4-c, P1, and T7) were visualized by TapeStation 4200 with genomic ScreenTape. **D** Percentage of dsDNA phage genomes with T4 or T4-c generated by different library preparation methods. Different colors indicate different phage genomes with equal input (11.11%). MG, Mock G contains high modification dsDNA T4 genome (T4) with equal ratio of all genomes; MH, Mock H contains lower modification dsDNA T4 genome (T4-c) with equal ratio of all genomes (Fig. S1G)

Notably, the dsDNA T4 genome, in which all cytosines are modified to 5-hydroxymethylcytosine (5-HMC) and further glucosylated (glc-HMC) [36], showed a distinct coverage pattern when prepared with MDA-related methods. The sequencing depths at all genome positions were quite low regardless of the MDA time (Fig. S3). This finding prompted further investigation by introducing an unmodified C-sites of T4-c genome, which instead have amber mutations in dCTPase and dHMase genes [37]. Quantification of the dsDNA T4 genome by NanoDrop showed significantly higher concentration (3.03–3.45 folds) than Qubit measurements of the same samples, while the less modified T4-c genome showed less differences (1.65-fold, Fig. 3A). MDA amplification of T4 genome resulted in relatively lower amount of product with shorter fragments compared to dsDNA phage genomes P1, T7, and T4-c (Fig. 3B and C). Equal amounts of phage genome (11.11% of each, quantified by NanoDrop) were used for preparation of Mock G (with T4) and Mock H (with T4-c), and we observed that dsDNA T4-c genome still yields lower percentage after sequencing (Fig. S6) but had higher ratios (about twofold) than T4 in Nextera, SSLR, and xGen method (Fig. S1C, Fig. S1G, Fig. 3D, and Table S5).

## SSLR can simultaneously identify DNA and RNA viruses

**Fig. 4:**
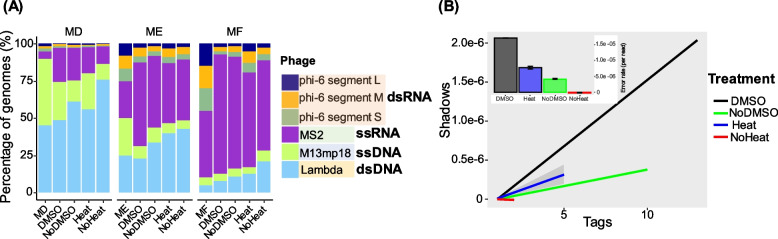
Comparison of the efficiency of SSLR on the simultaneous identification of DNA/RNA mock communities. SSLR was used to prepare and sequence four artificial virome containing different proportions of the DNA phage and RNA phage. **A** Effect of DMSO and heat treatment on the percentage of four phage genomes. These phage genome abundance values were calculated based on the quantity of DNA and RNA phages measured by NanoDrop. MD, Mock D with ratio of 90:10 for DNA and RNA; ME, Mock E with ratio of 50:50 for DNA and RNA; MF, Mock F with ratio of 10:90 for DNA and RNA. phi6 has 3 segments: large (6374 bp), medium (4063 bp), and small (2948 bp). **B** Heat treatment does not adversely affect sequencing error rates. The R package ShadowRegression estimates reference-free error rates (inset) based on a transform of the slope of read counts and their “shadows” (main plot line graphs). Shadows (*y*-axis) are a measure of the variation in read counts across different sequencing runs for the same sample. They are calculated by taking the logarithm of the ratio of read counts in one run to another run. A higher shadow value means a larger difference in read counts between the two runs. Tags (*x*-axis) are a measure of the abundance of reads for a given nucleotide position in a sample. They are calculated by taking the logarithm of the read count at that position. A higher tag value means a higher number of reads at that position. The figure shows the relationship between shadows and tags for different samples treated with or without heat/DMSO. The slope of this relationship is used to estimate the sequencing error rate for each sample, which is shown in the inset plots. The figures suggest that DMSO treatment does not affect the sequencing error rate significantly

Next, we applied the SSLR method for simultaneous sequencing of DNA and RNA phage genomes (Table 1, Fig. 1, Fig. S1B and F). We used DMSO or heat treatment to denature the dsRNA to facilitate the reverse transcription (RT) process. We found both treatments including the control (No DMSO) could increase the ratio of ssRNA compared heat or no heat, especially for the low ssRNA mock (5% of ssRNA, Mock D) (Fig. 4A and Fig. S7). Although pretreatment with DMSO increased the number of reads of dsRNA compared to non-DMSO treatment in the mocks with less dsRNA (5 ~ 25% of dsRNA, Mock D and E, Table S6), it was not as effective as heat treatment in terms of read accuracy and sequence coverage (Fig. 4B, Fig. S7, and Table S6).

**Table 1:** Overview of phage genome characteristics included in the mock communities from the present study

Phage	Family (species)*	Structure	Genomic type	Genomic topology	Number of genomic segment(s)	Genomic length (bp)	GC%
C2	**(*Ceduovirus* c2)	Non-enveloped	dsDNA	Linear	1	22,172	36.31%
T4 (T4-c)#	Straboviridae (*Tequatrovirus* T4)	Non-enveloped	dsDNA	Linear	1	168,903	35.30%
Phi29	Salasmaviridae (*Salasvirus* phi29)	Non-enveloped	dsDNA	Linear	1	19,282	40.00%
P1	**(*Punavirus* P1)	Non-enveloped	dsDNA	Linear	2	94,800	47.31%
T7	Autographiviridae (*Teseptimavirus* T7)	Non-enveloped	dsDNA	Linear	1	39,937	48.40%
P35	(*Listeria* phage P35)	Non-enveloped	dsDNA	Linear	1	35,822	40.82%
Lambda	**(*Lambdavirus* lambda)	Non-enveloped	dsDNA	Linear	1	48,502	49.86%
PhiX174	Microviridae (*Sinsheimervirus* phiX174)	Non-enveloped	**ssDNA (> 85%)**	**Circular**	1	5386	44.76%
M13mp18	Inoviridae (*Inovirus* M13)	Non-enveloped	**ssDNA**	**Circular**	1	7249	42.27%
MS2	Fiersviridae (*Emesvirus* MS2)	Non-enveloped	**ssRNA**	Linear	1	3569	52.12%
Phi6	Cystoviridae (*Cystovirus* phi6)	**Enveloped**	**dsRNA**	Linear	3	13,385	55.82%

^*^Current taxonomy of ICTV217

^**^No family levels

^#^T4-c has the same genome characteristic as T4 but with heavily modified groups

## SSLR provides high-quality fecal virome characterization and allows detection of highly modified phage genome

**Fig. 5:**
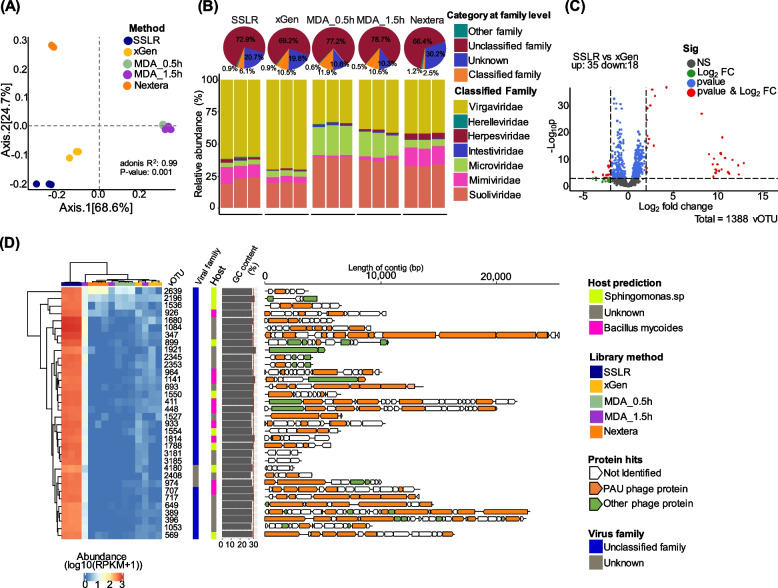
Comparison of different library strategies for fecalvirome communities. **A** Principal coordinates analysis (PCoA) plots of Bray–Curtis distance matrices. PCoA was used to plot the beta diversity of viral-associated communities using the bray matrix. Different colors indicate different library preparation methods. For each axis, in square brackets, the percentage of variation explained was reported. **B** Representative taxonomic distribution (relative abundance) of the sequenced virome. Top pie chart shows the the percentage of the classified, unclassified and unknown at the taxa of family level. The bottom bar chart only includes the classified taxa at the taxonomical level of the family. The taxonomy of contigs was determined by querying the viral contigs against a database containing taxon signature genes for virus orthologous group hosted at www.vogdb.org. The unclassified are the contigs that cannot be assigned to any known viral taxonomy at the family level; the unknown is the contigs that are related to “viral dark matter.” **C** The volcano plot shows differentially abundant vOTUs identified from DESeq2 analysis, displaying those with a fold change of two or greater when comparing SSLR to xGen. Each dot represents a vOTU contig and is colored to indicate significance. Gray, not significant (NS); green, significant by log2 fold change (> 2); blue, significant by *p*-value (< 0.01); red, significant by log2 fold change and *p*-value. **D** Heatmap of the high abundance (RPKM) viral contigs that were highly enriched in SSLR method that does not present in the rest of methods. This figure shows a subset of the data presented in Fig. S9A. Different colors indicate different annotated protein, and directional boxes indicate open reading frames (ORFs) in the respective orientation. Non-filled arrows indicate no protein hits, and the rest of the colored arrows are hits to the majority of PAU proteins

Finally, we tested the capability for determining fecal virome composition using the methods described above (Fig. 1 and Fig. S1D). Although both MDA-based methods yielded more reads that can be assigned to reference viral database (RVDB) (range, 5.77–6.26%, Fig. S8A and Table S7) compared to SSLR (0.83–1.20%) and xGen (1.23–1.38%) (Fig. S8B), 96% of viral operational taxonomic units (vOTUs) were shared among different methods (Fig. S8C). We also observed that although the MDA method resulted in higher alignment rates, it led to lower virome Shannon diversity compared to the other library preparation methods (Fig. S8D and E). And for both short- and long-time MDA, proportions of ssDNA viral families were increased compared to other library preparation methods (Table S8). The SSLR could capture around two times less of single genome virome (ssDNA of Microviridae and ssRNA of Virgaviridae) than xGen method (Table S8, Fig. 5B, and Fig. S8F).

Additionally, the different library preparation methods led to differential abundant core species as compared with the Nextera method, although the majority of vOTUs are not identified at family level (Fig. S8F). Based on the virome abundance (log10 (RPKM + 1)), four clusters were identified (Fig. S9A). Notably, the abundance of cluster 3 from the SSLR method was approximately 10^3^ times higher than the other methods (Fig. 5D and Fig. S9B). We found that these 34 vOTUs were associated with multiple hits to *Sphingomonas* phage PAU (previous Myoviridae) (Fig. 5C, D and Fig. S9C). As it is evident that the cytosines in *Sphingomonas* phage PAU genome were highly modified [38], this further confirmed that SSLR method can efficiently include modified viral genome in the sequencing libraries.

## Discussion

The viral community in the human gut is highly complex and likely plays an important role in maintaining gut homeostasis [1, 39]. However, whether the true diversity and distribution of gut virome can be accurately determined by traditional nucleic acids library construction and sequencing methods and with what kind and degree of biases are only partly known. In this study, we addressed these problems by introducing a series of phage genome mock communities reflecting different ratios of four types of viral genomes (dsDNA, ssDNA, dsRNA, ssRNA). To ensure accuracy and avoid extraction efficiency variations [40, 41], we used phage genome (DNA/RNA quantified by NanoDrop or Qubit) as initial inputs instead of phage particles by plaque assay or SYBR green counts. The single-stranded library preparation method (SSLR) we introduced for gut virome studies proved to be highly accurate, less biased, and comparably efficient, with the added benefits of being fast and cost-efficient.

SSLR demonstrated excellent efficiency in quantifying both dsDNA and ssDNA genomes, overcoming the major limitation of the Nextera protocol, which only works for dsDNA genomes [7, 42]. While MDA can be applied for turning ssDNA into dsDNA before using Nextera, and it is known for its amplification preference for ssDNA genomes [23–25]. In line with the previous studies [24], we showed that MDA led to two- to threefold overestimation of ssDNA in low ssDNA mocks (Mock A, around 10% of ssDNA) but not in high ssDNA genome mocks (≥ 50%, Mock B and C). Additionally, the amplification bias appeared to depend on the type of ssDNA genome, as MDA preferentially amplified the alpha3 genome more than PhiX174 and M13mp18, which might be associated to the circular ssDNA of alpha3 being amplified faster than linear ssDNA or the different responses of ssDNA genomes to the heat-denaturing before MDA [23]. Similar to previous studies, we also found that SSLR and xGen slightly underestimated ssDNA in mocks with a low ratio of ssDNA (≤ 10%); this might be due to the ssDNA being more sensitive to shearing during the sonication step compared to dsDNA [24, 43].

T4 is an exception among dsDNA genomes in the mocks, as its relative abundance in SSLR and xGen was consistently higher than its input (1.6- to 3.7-fold) and higher than its abundances in Nextera (1.96- to 9.59-fold) and MDA (99–586 folds). Interestingly, we observed a significant and consistent difference between the quantification of DNA concentration by Qubit and NanoDrop when measuring T4 genomes. This discrepancy may be attributed to glucosylated 5-hydroxymethyl-cytosine (glu-5HMC) modifications present in the T4 genome [36, 37]. These modifications could interfere with or alter the binding modes of the Qubit dye, leading to lower concentration values than those obtained through UV absorbance-based quantification (NanoDrop) [44]. Considering the high quality of our T4 genome, which was extracted, purified, and recovered from agarose gel (see the method part), the concentration values measured from NanoDrop should be more accurate, as three times concentration higher from nanodrop is corresponding to the ~ 3-time higher sequencing output (Fig. 3A vs Fig. 2A). Our results also shed light on the reason why the 7-deazaguanine-modified *Cellulophaga* phage phi38:2 (previous Myoviridae) was overestimated in the A-LA method (xGen) and underestimated in MDA [24], as it was demonstrated recently that this phage has extensive modifications on its genome to protect its DNA from bacterial defense systems [45]. It was also reported that when measuring potentially highly modified viral DNA using DNA-binding fluorescent dyes, greater caution should be exercised to avoid pitfalls [46]. In comparison to the T4 genome, the T4-c genome is less modified, and its genome can be sequenced deeper using the Nextera method (Fig. S6). However, in the MDA-related method, the genome yield was not increased for T4-c suggesting the remaining modifications in T4-c genome could still inhibit the MDA process on a similar level.

SSLR has an advantage for sequencing highly modified viral genomes (such as dsDNA T4 with 5-HMC) compared to existing methods, which is further demonstrated by the detection of dsDNA PAU phage in the fecal virome. We failed to identify PAU-like phage from four gut virome databases (GVD, GPD, MGV, and IMG_VR4.1, Table S10) [47–50], probably due to the modified phages which were not included in the original metagenomic sequencing libraries. This finding suggested that many virome studies may have missed or at least underestimated the existence of viruses with highly modified genomes.

Unlike the widely used Nextera library preparation method, which can be affected by genome size and GC content [51, 52], the proposed SSLR protocol exhibits comparable alignment rate and low read errors and provides comprehensive ability for variable genomes with exceptional evenness of coverage and near-complete assembly of phage genomes, as also exemplified for the otherwise “difficult” phage T4 (Table S11). We also observed that SSLR outperforms xGen in several performance metrics (including assembly quality, evenness of genome coverage, and error rate (Fig. S3 and Table S4)) probably due to the fact that it does not need synthesizing a second strand for ssDNA viral genomes prior to adaptor ligation, thus retaining the native termini and avoiding possible artifacts or errors presented during sequencing library preparation [35].

SSLR also offers the advantage of using cDNA directly, the first-strand products of RT, as ligation templates for sequencing of RNA viruses. Unlike NetoVIR and FLDS, the SSLR simplifies the RT step and preserves the strand information [34, 35]. Our DNA and RNA genome mocks tests demonstrated that the RT step did not significantly affect the distribution of DNA viruses, although the ssRNA virus MS2 was overrepresented. This overrepresentation can be attributed to the faster RT conversion rate of ssRNA compared to dsRNA, leading to nonuniformity in read coverage, which is consistent with previous observations [53]. Furthermore, for denaturing of dsRNA, the heat-shock treatment appears to be a more cost-effective and easier approach compared to DMSO treatment, and it not only reduces the denaturation time but also eliminates the necessity to remove DMSO after treatment, thereby conserving more sample [53, 54].

In conclusion, we introduce an improved, rapid, straightforward, and efficient ligation-based single-stranded DNA library preparation method tailored for virome studies. Additionally, the SSLR may also be a good tool for metagenomics library preparation, in which it can capture both virome and bacteriome with a minimal bias regardless of their genome types, but this still needs to be verified. We believe that the flexibility and adaptability of our approach can contribute to the advancement of viromics across various fields, offering valuable insights into viral ecology and evolution.

## Materials and methods

### Mock communities with phage DNA and RNA genomes

Customized bacteriophage mock community samples were designed and prepared, comprising of a mixture of 4 to 9 bacteriophage genomes with different genome sizes (3.5 to 168.9 kbp), genome types (dsDNA, ssDNA, dsRNA, and ssRNA), G + C content (35 to 56%), the presence or absence of an envelope, and different genome compositions (linear, circular, segmented) (Table 1). The phages were propagated using the conditions listed (Table S10), and the genomic DNA/RNA extractions were conducted using commercial extraction kits (DNeasy Blood and Tissue Kit for DNA or RNeasy Mini Kit for RNA, both from Qiagen) and purified with GeneJET Genomic DNA Purification Kit (Thermo Scientific, no. K0722) for recovering the high-quality genome. The quality and integrity of DNA and RNA were checked by agarose gel electrophoresis and Agilent TapeStation 4200 High Sensitivity (HS) RNA ScreenTape. Their concentrations were quantified by dsDNA (or ssDNA) HS Assay Kit or RNA HS Assay kit (Thermo Fisher Scientific, Waltham, MA, USA) with Qubit 4.0, along with NanoDrop1000 (V3.8.1).

## DNA phages library preparation and sequencing

For the DNA phage library preparation, we mixed genomic DNA of nine phages (seven dsDNAs and two ssDNAs) with different ratios (Mock A, Mock B, and Mock C) to test five different library preparation methods (Fig. 1, Fig. S1A, Fig. S1E, and Table S1).

Nextera XT libraries (Nextera) were prepared according to the manufacturer’s protocol (FC-131–1096) (Illumina). Briefly, 1.0 ng of each DNA library was enzymatically fragmented and tagged by tagmentation. Amplification was performed using Illumina dual index (i7 + i5) adapters and cleaned by AMPure XP bead cleanup (A63880l; Beckman Coulter). Two technical replicates were generated for each biological sample, resulting in six Nextera-prepared libraries.

The same mixture of phage DNA as mentioned above was amplified by multiple displacement amplification (MDA) using the GenomePhi V3 kit (GE Healthcare Life Science, Marlborough, MA, USA) with 10-ng input and incubated at 30 °C for 0.5 h (MDA_0.5 h) or 1.5 h (MDA_1.5 h). Subsequently, the amplified DNA was cleaned using Genomic DNA Clean & Concentrator™ kit (Zymo Research, Irvine, CA, USA). Qubit® 1X dsDNA HS Assay Kit on Qubit Fluorometer (Life Technologies, CA, USA) was used to measure the concentration of the purified DNA. Finally, the library was constructed using the Nextera XT kit (Illumina, San Diego, CA, USA). Two technical replicates were generated for each biological sample, resulting in a total of 12 MDA-prepared libraries.

xGen™ ssDNA and Low-Input DNA Library (xGen, previously known as Swift Bioscience Accel-NGS® 1S) was prepared following the manufacturer’s protocol (Catalog no. 10009859, Integrated DNA Technologies, IDT). The Mock (15 ng in 20-µl TE buffer) was randomly fragmented by Bioruptor (Diagenode, Liege, Belgium) at default intensity for 15 s on and 90 s off, with seven cycles at 4 °C as described by Hoang et al. [55]. Sheared DNAs were firstly subjected to 3′-end tailing and ligation with adapter1, followed by extension and ligation with adapter2. The purified ligation products were indexed with xGen™ unique dual index (UDI) primers (Catalog no. 10005975, IDT) and amplified through a 10-cycle PCR. The amplified products were cleaned up with AMPure XP beads (Beckman Coulter Genomic, CA, USA). For each biological sample, two technical replicates were conducted, resulting in a total of six xGen-prepared libraries.

For the single-stranded library preparation (SSLR, detailed in Supplementary file 1), the same mocks were used. After random fragmentation, the end-modified adapters (Supplementary file 2) synthesized by IDT (IDT, Leuven, Belgium) were ligated to DNA fragments employing the modified method from Troll et al. [35], and a detailed method can be found in Supplementary file 1 or protocol.io (dx.doi.org/10.17504/protocols.io.8epv5r5w5g1b/v1). Subsequently, the ligation mix was purified using MinElute Reaction Kit (Qiagen, Hilden, Germany), and the ligation products were eluted with 23 µl of TE buffer (10 mM, pH8.0). The cleaned ligation products were then amplified and indexed for sequencing with Illumina dual indexes (i7 + i5) and further purified using AMPure XP beads (Beckman Coulter Genomic, CA, USA). An additional bead cleanup step can be applied to mitigate the formation of byproducts and adapter dimers resulting from self-ligation of the adapters (Fig. S10). Two technical replicates were performed for each biological sample, resulting in a total of six SSLR-prepared libraries. A detailed protocol can be found on Supplementary file 1.

The DNA concentrations of Nextera-prepared, MDA-prepared, xGen-prepared, and SSLR-prepared libraries were measured by Qubit® 1X dsDNA HS Assay Kit on Qubit Fluorometer (Life Technologies, CA, USA), and fragment length distributions were determined using TapeStation 4200 (Agilent, CA, USA). Equal amounts of DNA from each library (except xGen prepared) were pooled and sequenced using 2 × 150 bp paired-end settings on an Illumina NextSeq 550 platform (Illumina, CA, USA). The xGen-prepared libraries were sequenced separately on an iSeq100 System (Illumina, CA, USA) using 2 × 150 bp paired-end settings due to their indexes being incompatible with the Illumina index.

## RNA phages library preparation and sequencing

Four types of phage genome (Table 1, dsDNA, ssDNA, dsRNA, and ssRNA) with different ratios were prepared for phage DNA/RNA mock communities (Mock D, Mock E, and Mock F). We used three mock communities to determine the applicability of the SSLR method for quantification of RNA and DNA phages simultaneously (Table 1, Fig. S1B and F). Specifically, 20-µl mock community was treated with DMSO at a final concentration of 50% and incubated at 65 °C for 90 min [53]. The DMSO was subsequently removed using a QIAmp Viral RNA Mini Kit (Qiagen, Hilden, Germany), according to the manufacturer’s instructions. As a control, a DMSO-free treatment was included. Alternatively, the same mock community was denatured at 95 °C for 3 min and immediately snap-cooled on ice, maintaining the nucleic acids as single stranded. Again, a sample without heat treatment was included as a control library.

All the DMSO-treated samples, heat-treated samples, and their corresponding controls were subjected to reverse transcription in a 20-µl reaction system according to the user guide (SuperScript™ IV VILO™ Master Mix, Invitrogen™), and the reaction mix was purified using AMPure XP beads, and a 20 µl of Tris buffer (10 mM, pH 8.0) was used for the final elution. Finally, the library preparations with SSLR were performed according to the abovementioned steps and sequenced on a NovaSeq 6000 platform (Illumina, CA, USA).

## Sequencing of highly modified phages

Two types of DNA phage mock communities (Mock G and Mock H) were prepared with equal input (11.11% for each of the nine genomes, Fig. S1C and G). Mock G contained a highly modified T4 genome (T4), while Mock H contained a less modified T4-c genome (T4-c). All the libraries were prepared as described above and sequenced with the NovaSeq 6000 platform (Illumina, CA, USA).

## Human fecal virome isolation and sequencing library preparation

For environmental sample, a fresh fecal sample was obtained from an anonymous healthy adult (Ethical Committee of the Capital Region of Denmark registration number H-20028549) and thoroughly mixed with SM buffer. Fecal virome isolation and purification were carried out according to our previous method with the following modifications [16]: the Centriprep 50 K was replaced by Centrisart® I centrifugal ultrafiltration unit (MWCO 100 kDa, Sartorius Stedim Biotech GmbH), and the enrichment step was conducted at 2500 × g for 30 min at 4 °C. The detailed protocol for this can be found here: https://www.protocols.io/view/vlp-extraction-from-fecal-samples-3byl4ko48vo5/v2. The QIAmp Viral RNA Mini Kit (Qiagen, Hilden, Germany) was used for the extraction of viral DNA/RNA from the concentrated virome solution (Fig. S1D). The library preparations were conducted with the aforementioned methods in triplicate. An equal amount of DNA from each library was pooled and sequenced using 150-bp paired-end settings on an NextSeq 550 platform (Illumina, CA, USA).

## Metavirome sequencing, data preprocessing, and data analyses

Metavirome reads were quality filtered, trimmed, and assembled using a previously published pipeline from GitHub: https://github.com/jcame/virome_analysis‑FOOD. Detailed data analyses for sequencing of the mock communities and fecal virome can be found from Supplementary file 3. The percentage of phage genomes was calculated by aligning the clean reads to customed database (https://github.com/XC-Zhai/SSLR/tree/main/Customed_db) with bowtie2 to assess the relative abundance of each DNA phage (Table S6). For mock community data, plots were generated using the ggplot2 [56] package in R (v4.2.0). Analyses of viral community *α*- and *β*-diversity were performed using packages phyloseq (v1. 36.0) [57] and vegan (v2.6.2) [58] in R. For *α*-diversity analyses, indexes of observed taxa and Shannon diversity were calculated with *t*-test using the package ggsignif (v0.6.3) [59]. For *β*-diversity analyses, Bray–Curtis distance metrics were calculated, and unconstrained ordination was performed using principal coordinate analysis (PCoA). To identify differentially enriched viral operational taxonomic units on the summarized family level, DESeq2 (v1.42.0) was adopted [60]. The results were then visualized in a heatmap using the R package ComplexHeatmap (v2.18.0) [61]. Estimation of sequencing error rates was conducted according to Wilcox et al. [53]. Coverage biases were visualized using the Python script from https://github.com/padbr/gcbias, with modifications made by adding the Pearson correlation. Genomic maps of the open reading frames (ORFs) which are predicted by prodigal and then annotated by the blast from the NCBI protein database, the best hits, were used for the annotation. To visualize the functional genes of annotated viral contigs, DnaFeaturesViewer (v3.1.0) [62] and R package gggenomes (v 1.01) [63] were used.

## Supplementary Information

Additional file 1. Step-by-step library preparation protocol for the reproposed SSLR.

Additional file 2. SSLR adapter design.

Additional file 3. Metavirome sequencing and data pre-processing.

Additional file 4. Supplementary Table S1-S11. TableS1: Summary of DNA genome sequences. Top table: Relative abundance and coverage of phage genomes in libraries from DNA mock communities (Mock A, Mock B and Mock C) with T4 genome. The relative abundance of each genome was calculated based on its coverage by using bowtie2 alignment with our customed database (https://github.com/XC-Zhai/SSLR/tree/main/Customed_db). For comparing the efficiency of different methods for each group of phages (i.e., dsDNA and ssDNA), relative abundance of two type genomes were computed from this table (by dividing the value for each phage by the sum for the group). Middle table: The average coverage for each genome from each library preparation method. Bottom table: similar to top table without T4 genome. TableS2: Comparisons of cost and effectiveness of Mock virome library preparation method. This comparison only considers the library preparation step for DNA virome. TableS3: Overview of metavirome sequencing for the DNA phage mock community. Statistics from assembly before and after quality checks with CheckV, vibrant and virsorter2. Contigs from each library prepared methods were evaluated by quality checks with CheckV, vibrant and virsorter2 and then subjected to the Quast for quality assessment of assembled contigs. The theatrical statistics of mock community were also subjected to Quast and bolded at the bottom of table (in yellow background). TableS4: Summary of contigs hits to the customed mock database. The quality-checked contigs were subjected to the customed database and the hit number was counted and listed in the table. TableS5: Summary of DNA genome sequences with interested modified genomes (T4 and T4-c). T4 is a highly modified genome, T4-c has lower modification compared to T4 genome. TableS6: Summary of DNA/RNA genome sequences prepared with SSLR method. Relative abundance and coverage of phage genomes in libraries from DNA/RNA mock communities (Mock D, Mock E and Mock F) with different treatments (heat, no heat, DMSO and no DMSO). The relative abundance of each genome was calculated based on its coverage by using bowtie2 alignment with our customed database. For comparing the efficiency of different treatments for each type of phage genome (DNA and RNA), relative abundance of two type genomes (DNA or RNA) were calculated from this table (by dividing the value for each phage by the sum for the genome types). Middle table: The average coverage for each genome from each library preparation method. The average coverage for each genome from each treatment method is listed in the bottom table. TableS7: Taxonomic classification of fecal metavirome sequencing reads into the categories viral, human, bacterial, and unknown origin. To check the presence of non-viral DNA sequences, 50,000 random forward reads were used according to their match to a range of viral, bacterial, and human reference database of Kaiju 1.8.2. TableS8: Relative abundance of fecal virome prepared with 5 different library strategies at taxonomy of family level (support data for Fig.5B) and the overall alignment rate of different library preparation methods based on bowtie2 alignment to the vog217 database. TableS9: Top 5 hits of PAU phage on 4 virus databases (GVD, GPD, MGV and IMG_VR4.1). TableS10: Characteristics of phage genomes included in the mock communities from the present study, as well as growth conditions for the strains in the mock communities. TableS11: Average alignment rate of each library to the customed databases for all the mock communities tested in the present study.

Additional file 5. Supplementary Figures S1-S10. Fig. S1 | Virome sequencing library preparation strategy with different types of nucleic acids (dsDNA, ssDNA, dsRNA and ssRNA). (A) Workflow for DNA phage genomes (dsDNA and ssDNA) library preparation with different library methods to varying ratios of genomes (Mock A, Mock B and Mock C). (B) Workflow for DNA and RNA phage genomes library preparation with the single-stranded library at different ratios of genomes (Mock D, Mock E and Mock F). (C) Workflow for comparison of the modification existence for (Mock G with T4, Mock H with T4-c), where T4 is a heavily modified genome and T4-c is a lower modified genome. (D) Workflow for comparison of the fecal sample (Fecal virome: FV) with different library preparations. (E) Percentage of phage genomes of DNAs library with different ratios of ssDNA and dsDNA phage genomes, three artificial virome communities (Mock A, Mock B and Mock C) containing different proportions of the ssDNA phage phiX174 and M13mp18 mixed with dsDNA phage. These phage genome abundance values were calculated based on the quantity of dsDNA and ssDNA phages measured Qubit dsDNA (or ssDNA) HS Assay kit. Mock A: ratio of ~90:10 for dsDNA and ssDNA; Mock B: ratio of ~50:50 for dsDNA and ssDNA; Mock C: ratio of ~50:50 for dsDNA and ssDNA. (F) Percentage of phage genomes of DNA/RNAs library with different ratios of DNA and RNA phage genomes, three artificial viromes containing different proportions of the dsRNA phage Phi6 and ssRNA MS2 mixed with DNA phage. These phage genome abundance values were calculated based on the quantity of DNA and RNA phages measured with Nanodrop1000. Mock D: ratio of 90:10 for DNA and RNA; Mock E: ratio of 50:50 for DNA and RNA; Mock F: ratio of 10:90 for DNA and RNA. (G) Percentage of phage genomes of DNAs library with 2 different T4 genomes, where MG: Mock G containing high modification T4 genome (T4) with equal ratio of all genomes; MH: Mock H containing lower modification T4 genome (T4-c) with equal ratio of all genomes. Fig. S2 Pearson correlation coefficient (r) and two-tailed p-value between the expected and obtained read distributions (in percentage) in different DNAs phage mock communities with or without phage T4 genome. (A) Pearson correlation only dsDNA phage genome with (Top panel) or without T4 (bottom panel) shows that the presence of T4 distorted the percentage of dsDNA phage genomes. The phage genome size (B) and GC_content (C) have minimal effect on the phage genome distributions for the library prepared with SSLR and xGen. Fig. S3 Different library methods induce different sequence coverages of the DNA phage genomes. Mock A was chosen for coverage visualization as it contains a high ratio of DNA genomes compared to Mock B and C. Read depth at each position in the phage genome was normalized by count/total reads*10000 for easier comparison among different library preparation methods. T4 genome amplified by MDA for 0.5 and 1.5 h resulted in low coverages. Fig. S4 (A) The overall percentages of sequencing reads mapping to the 9 DNA reference genomes. The R package ShadowRegression estimates reference-free error rates (B) based on a transform of the slope of read counts and their ‘shadows’ (C). (D) Depth biases in the sequencing of DNA phage genomes prepared with different library methods. The circle plot shows from the inside: Nextera (Ring 1); MDA_0.5h (Ring 2); MDA_1.5h (Ring 3); xGen (Ring 4) and SSLR (Ring 5). The circles are numbered from the inside. The sequencing depth was shown in the log10 scale. All the reads were normalized and then visualized using the script from https://github.com/padbr/gcbias. Fig. S5 Coverage biases in datasets for the different DNA phage genomes with different GC contents. Dot plots show local GC content and normalized relative coverages in 50-nt windows of NextSeq/iSeq and data from a variety of phages with different average GC contents. Error bars indicate ±1 standard deviation of normalized coverage. The intensity of the blue in the dots is a log-transformed heat map of the number of 50-nt windows averaged into that data point. The data point with the most windows in each plot has maximum red. The vertical green line marks the average GC content of each assembly. The average normalized coverage value is indicated with a horizontal dashed red line. The plots were visualized with the Python script from: https://github.com/padbr/gcbias, with the modifications by adding the Pearson correlation coefficient (r), and two-tailed p-value (top right corner of each plot). Fig. S6 Different library methods induce different sequence coverages of the T4 (blue line)/T4-c (purple line) phage genomes. Read depth at each position in the phage genome was normalized by count/total reads*10000 for easier comparison among different library preparation methods. Fig. S7 (A) Effect of DMSO and heat treatment on sequence coverage of the RNA phage genomes. Read depth at each position in the phage genomes under varying treatments (DMSO and Heat) and their controls (NoDMSO and NoHeat) are shown. (B) Coverage biases in datasets for the different types of RNA phage genomes with different GC contents. Dot plots show local GC content and normalized relative coverages in 50-nt windows and data from a variety of phages with different average GC contents. Error bars indicate ±1 standard deviation of normalized coverage. The intensity of the blue in the dots is a log-transformed heat map of the number of 50-nt windows averaged into that data point. The data point with the most windows in each plot has maximum red. The vertical green line marks the average GC content of each assembly. The average normalized coverage value is indicated with a horizontal dashed red line. The plots were visualized with the Python script from: https://github.com/padbr/gcbias, with the modifications by adding the Pearson correlation coefficient (r), and two-tailed p-value (top right corner of each plot). (C) Pearson correlation coefficient (r) and two-tailed p-value between the expected and obtained read distributions (in percentage) in different DNA/RNA phage mock communities. The phage genome size (D) and GC_content (E) have minimal effect on the phage genome distributions for the library prepared with SSLR. Fig. S8 | (A) Distribution of sequencing reads into the different taxonomic categories as viral, human, bacterial, fungi, and unknown origin. To check the presence of non-viral DNA sequences, 50,000 random forward reads were evaluated according to their match to a range of viral, bacterial, and human reference genome and protein databases. No reads (in 50,000 reads) matched the 18S rRNA gene sequences in all the samples. (B) The ratio of viruses and unknown parts of fecal samples prepared with different methods. (C) The Venn diagram indicates the presence of vOTUs in the different library preparation methods. (D) The overall alignment rate (%) of high-quality contigs that mapped to viral ref database. (E) The effect of different library preparation methods on the viral overall-alpha diversity with the measurement of Observed and Shannon index. NS indicates not significant, one asterisk indicates a significant difference at p < 0.05 (t.test), two asterisk indicates a significant difference at p < 0.01 (t.test), three asterisk indicates a very significant difference at p < 0.001 (t.test). (F) Differential abundance analysis by comparing the tested library preparation methods on the distribution of viruses at the family level (Nextera XT library method was used as reference). Hierarchical clustering was performed to group family (rows) and library preparation methods (columns) based on similarities in differential abundance signatures and was visualized via heatmap. Fig. S9 (A) The heatmap shows the abundances (log10(RPKM+1)) of fecal virome contigs, and the virome contigs were divided into 4 clusters. (B) The abundances of the 4 divided clusters. (C) 34 of high abundance (RPKM) viral contigs that were highly enriched in the SSLR method that are not present in the rest of the methods. Contig was piped through VirSorter 2 and DRAMv for viral identification and annotation. Multiple hits matched to Sphingomonas phage PAU (NC_019521.1). Fig. S10 Library size assessment by Agilent TapeStation 4200. (A) The extra clean-up step can remove the dimers that the first-round bead clean-up cannot remove. The fecal virome library was prepared by the SSLR and cleaned up by 0.8x AMPure XP beads. (B) Different library methods result in different library size distributions. In general, both MDA and Nextera XT tend to have shorter library sizes compared to single-stranded methods (SSLR and xGen).

## Data Availability

The authors declare that the data supporting the findings of this study are available within the paper and its supplemental information files. All the raw viral metagenome sequences data produced in this study are available through the National Center for Biotechnology Informationʼs Sequence Read Archive under BioProject accession number PRJNA1094595 with submission no. SUB13375064 (DNA mocks), no. SUB14350434 (DNA/RNA mock), no. SUB14350532 (modification mock), and no. SUB14350824 (fecal virome). Code for metavirome pre-analysis is available from GitHub: https://github.com/jcame/virome_analysis-FOOD. Original Python code from (https://github.com/padbr/gcbias) with modifications for fitting the GC bias can be found from GitHub: https://github.com/padbr/gcbias. The read accuracy and mutation analysis can be found on GitHub: https://github.com/awilcox83/dsRNA-sequencing. The public databases for checkV (v1.5) at https://portal.nersc.gov/CheckV, VIBRANT (v1.2.1) at (https://github.com/AnantharamanLab/VIBRANT/tree/master/databases), Virsoter2 at (https://osf.io/u3t4j), VOG217 at (https://fileshare.csb.univie.ac.at/vog/vog217), and Iphop (v1.3.2) at (https://portal.nersc.gov/cfs/m342/iphop/db/) are accessible online.

## Abbreviations

SSLR: Single-stranded library
SRSLY: Single reaction single-stranded LibrarY
RT: Reverse transcription
MDA: Multiple displacement amplification
PCoA: Principal coordinates analysis
vOTUs: Viral operational taxonomic units
ORFs: Open reading frames
NS: Not significant
GVD: Gut virome database
GPD: Gut Phage Database
MGV: Metagenomic gut virus
IMG/VR4.1: High-integrated microbial genomes/viruses 4.1
HS: High sensitivity
UDI: Unique dual index
IDT: Integrated DNA Technologies
NetoVIR: Novel enrichment technique of VIRomes
FLDS: Full-length double-stranded RNA sequencing
