# Supplementary material for: A single-stranded based library preparation method for virome characterization
Source: Microbiome. 2024 Oct 24;12:219. doi: 10.1186/s40168-024-01935-5 (PMC11515303; doi:10.1186/s40168-024-01935-5)
Supplement: Supplementary file 2 — Additional file 1. Step-by-step library preparation protocol for the reproposed SSLR. [file 40168_2024_1935_MOESM1_ESM.pdf]

Sep 09, 2024

Single_Stranded_Library_Workflow_Gut_Virome

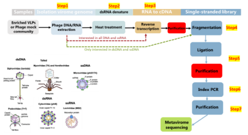

DOI
dx.doi.org/10.17504/protocols.io.8epv5r5w5g1b/v1

Xichuan Zhai[1]

[1]University of Copenhagen

Xichuan Zhai
University of Copenhagen

OPEN ACCESS

DOI: dx.doi.org/10.17504/protocols.io.8epv5r5w5g1b/v1

Protocol Citation: Xichuan Zhai 2024. Single_Stranded_Library_Workflow_Gut_Virome. **protocols.io** **https://dx.doi.org/10.17504/protocols.io.8epv5r5w5g1b/v1**

Manuscript citation:
https://doi.org/10.21203/rs.3.rs-4304844/v1

License: This is an open access protocol distributed under the terms of the **Creative Commons Attribution License**, which permits unrestricted use, distribution, and reproduction in any medium, provided the original author and source are credited

Protocol status: Working
We use this protocol and it's working

Created: September 08, 2024

Last Modified: September 09, 2024

Protocol Integer ID: 107129

Keywords: Single-stranded library preparation, gut virome

Funders Acknowledgement:
VILLUM FONDEN
Grant ID: 23145
VILLUM FONDEN
Grant ID: 36242

Abstract

Protocol of single-stranded library (SSLR) preparation for virome study

Guidelines

This protocol is designed for virome library preparation with our lab-customed single-stranded library (SSLR). This workflow includes **7 FLEXIBLE** steps, virome isolation and extraction, mock community (positive control) preparation, reverse transcription with the purpose of dsRNA denatures, genome fragmentation, ligation with customed designed adaptors and library preparation for sequencing. Detailed information can be found on the next page.

Materials

SM buffer: lab-prepared according to https://cshprotocols.cshlp.org/content/2006/1/pdb.rec8111.full?text_only=true
Pierce™ Universal Nuclease for Cell Lysis: #88701
QIAamp Viral RNA Mini Kit (250): # 52906
SuperScript™ IV VILO™ Master Mix: #11756050
AMPure XP beads, 60 mL: #A63881
TE, pH 8.0, RNase-free: #AM9849
ET SSB (500 µg/mL): #M2401S
T4 Polynucleotide Kinase (10 U/µL): #EK0031
T4 DNA Ligase (5 U/µL): #EL0012
T4 DNA Ligase Buffer (10X) with 50% PEG-4000: #B69
Forward/reverse adapters: Lab designed and produced by IDT
AccuPrime™ Taq DNA Polymerase System: #12339016
Nextera XT Index Kit v2 Sets: #FC-131-2001, 2002, 2003, 2004
Qubit 1X dsDNA HS Assay Kit: #Q33231
High Sensitivity D5000 ScreenTape Assay: #5067-5593

Protocol materials

Fresh 80% Ethanol — Step 7.7

Safety warnings

Not applied

Ethics statement

Not applied

Before start

Fill bucket with crushed ice.

Step1: Virome isolation and extraction

1 **Checklist before starting**

Note

Where: Fecal lab

Timing: 180 min

Reagents/kits
SM buffer: lab-prepared according to
https://cshprotocols.cshlp.org/content/2006/1/pdb.rec8111.full?text_only=true
PierceTM Universal Nuclease for Cell Lysis: #88701
QIAamp Viral RNA Mini Kit (250): # 52906

Equipment
Centrifuge, 37°C incubator

Procedure

1.1 Make aliquots of 140 µL of enriched virome (procedure can be found from: **dx.doi.org/10.17504/protocols.io.b2qaqdse**).

1.2 Add 1 µL of 100-time diluted nuclease (check the stock for the dilution in SM buffer) to each sample and let them incubate at approximately 00:30:00 at 37 °C per. sample. Vortex between each sample. 30m

1.3 Consider increasing the incubation time if you expect a lot of external DNA.

1.4 Immediately after adding 540 µL AVL buffer to inactivate nucleases.

1.5 Mix the mixture by pulse vortexing.

1.6 Incubate at Room temperature for 00:10:00 . 10m

1.7 Change gloves.

1.8 Briefly centrifuge the mixture with a microcentrifuge.

1.9 Add 560 µL of absolute ethanol (96%) to the sample mixture.

1.10 **CRITICAL STEP**: Mix very well by pulse-vortex.

1.11 Briefly centrifuge the mixture with a microcentrifuge.

1.12 Add 630 µL of the sample mixture to the spin column.

1.13 **CRITICAL STEP**: Do not touch the column rim with the pipet.

1.14 Centrifuge at 6,000 x g, Room temperature change the collection tube and repeat steps 1.12 to load all the extracts.

1.15 Add 500 µL AW1 buffer to the spin column the following:

1.16 Centrifuge at 6,000 x g, Room temperature, 00:01:00 , change the collection tube. 1m

1.17 Add 500 µL AW2 buffer to the spin column.

1.18 Centrifuge at 20,000 x g, Room temperature, 00:03:00 , change the collection tube. 3m

1.19 Centrifuge at 20,000 x g, Room temperature, 00:01:00 . Place the spin column in a low-binding RNAse-free 1.5 mL tube. 1m

1.20 Add 30 µL of AVE (elution buffer) to the spin column and incubate Room temperature for 00:01:00 . 1m

1.21 **CRITICAL STEP**: Pipet the AVE buffer directly onto the filter membrane without touching it with the pipet tip.

1.22 Centrifuge at 6,000 x g, Room temperature, 00:01:00 to collect the filtrates.

1m

Note

PAUSE POINT Send for next step or store viral DNA/RNA at -80°C.

CAUTION
NOT interested in **RNA**??? **THEN GO** go to step #4.
Interested in **DNA** and **ssRNA**, **THEN GO** go to step #3.
Interested in DNA, dsRNA and ssRNA, **THEN GO** go to step #2.
Remember to include positive (Mock from extraction) and Negative (H_2O from extraction) controls for each extraction.

Step2: Heat treatment

2 **Checklist before starting**

Note

Where: Clean-lab

Timing: 3 min

Reagents
No reagents needed

Equipment
UV-beach, Thermocycler, Microcentrifuge

Procedure

2.1 Preheated ThermoCycle machine to 95 °C and sterilized PCR tubes or plates depending on the
how many samples are used for reverse transcription.

2.2 Add 16 µL extracted virome in PCR tubes or platesand prepare on ice.

2.3 Briefly centrifuge the mixture with a microcentrifuge.

2.4 Put tubes or plates for heat treatment 95 °C for 00:03:00 .

3m

2.5 **CRITICAL STEP**: Transfer samples to ice immediately after 00:03:00 .

3m

2.6 **PAUSE POINT** Ready for reverse transcription.

Note

CAUTION
If you would like to look at the RNA in your samples, it is better to finish extraction, heat treatment, and reverse transcription **on the same day**.
Pipette, filtered pipette tips, and workbench need to be sterilized by UV lamp for 20 min prior work.
Remember to include Positive (Mock from extraction Step1) and Negative (SM buffer from extraction) controls for the reaction.

Step3: Reverse transcription (RT)

3 **Checklist before starting**

Note

Where: Clean-lab

Timing: 25 mim

Reagents/kits
SuperScriptTM IV VILOTM Master Mix: #11756050
AMPure XP beads, 60 mL: #A63881
TE, pH 8.0, RNase-free: #AM9849

Equipment
Thermocycler, Microcentrifuge, UV workbench

Procedure

3.1 Transfer 16 µL of extracted or heat-treated virome DNA/RNA to a clean PCR plate.

3.2 Add 4 µL SuperScriptTM IV VILOTM Master Mix to each sample and mix thoroughly, spin them down.

3.3 Carefully place the PCR plate into the ThermoCycle machine and select the following program:

A	B
Temperature profile	
25°C	10 min
50°C	10 min
85°C	5 min
4°C	∞

3.4 Purified with 1X AMPure XP beads (25 µL / 25 µL PCR reaction) and eluted with 20 µL Tris buffer (10 mM, pH8.0) according to the purification procedures from go to step #7.

Note

PAUSE POINT Samples can be stored in the fridge or freezer for downstream preparations.

Step4: Fragmentation

4 **Checklist before starting**

Note

Where: Basement (using ID card and key for access)

Timing: 15 min

Reagents
No reagent needed

Equipment
Bioruptor® Pico sonication device (#B01060010), Microcentrifuge

Procedure

4.1 **CRITICAL STEP**: Pre-cooled (4°C) the Biorupter and holder at least 00:30:00 . 30m

4.2 While waiting for cooling down, transfer genomic DNA or reverse-transcription products to a new, clean Bioruptor tubes and briefly spin down to make sure all the liquid is at the bottom of tubes.

4.3 Put samples on the ice for at least 00:10:00 . 10m

4.4 **CRITICAL STEP**: Set the sonication parameter as following: **15s ON and 90s OFF, using 8 cycles.**

4.5 **CRITICAL STEP**: Spin down the samples and carefully load the Bioruptor tubes to sonicater holder and close carefully, make sure there is no liquid at the side of tubes.

4.6 **CRITICAL STEP**: Put the tube holder into the sonication chamber and close the lids. 12 samples can be done each time.

4.7 Spin down the tubes after shearing.

4.8 The fragmented product is ready for ligation.

Note

PAUSE POINT Samples can be stored in fridge or freezer for downstream preparations.

CAUTION
Pregnant women should not stay away from the running machine.
DO NOT turn on the instrument without water.
Distilled water should be used to fill the tank.
Always keep 12 tubes for each run to ensure successful shearing.

Step5: Ligation

5 **Checklist before starting**

Note

Where: Clean-lab

Timing: 60 min

Reagents
ET SSB (500 µg/mL): #M2401S
T4 Polynucleotide Kinase (10 U/µL): #EK0031
T4 DNA Ligase (5 U/µL): #EL0012
T4 DNA Ligase Buffer (10X) with 50% PEG-4000: #B69
Forward/reverse adapters: Lab designed and produced by IDT
AMPure XP beads, 60 mL: #A63881

Equipment
Thermocycler, Microcentrifuge

Procedure

5.1 **Denature**

5.2 According to the number of samples (Include extra 3 for pipetting errors), prepare an ET SSB dilution in a clean tube. In the table below is calculated for a whole plate of 96 wells (Master Mix per 100 samples): diluted ET SSB to 5 ng/µL (500 µg/mL in the stock solution) with Tris and then add same volume of Tris, for example 1 µL ET SSB in 100 µL tris buffer. Mix them, spin them down and place on ice.

A	B	C
Reagent	Per 1 sample µL	Per 100 samples µL
Fragmented DNA	20	-
ET SSB dilution (10 ng)	2	100
Total denaturation reaction volume	**22**	

5.3 Add 20 µL of fragmented DNA to each well.

5.4 Add 2 µL ET SSB solution, pipette several times, and spin down.

5.5 Transfer to ThermoCycle and denature for 00:03:00 at 95 °C . 3m

5.6 Cooling down on ice immediately after heating and set On ice for at least 00:05:00 . 5m

5.7 **Ligation**

5.8 Spin down the heat denatured sample with a microcentrifuge.

5.9 According to the number of samples (Include extra 3 for pipetting errors), prepare a Master Mix containing per sample. In the table below is calculated for a whole plate of 96 wells (Master Mix per 100 samples). Mix them, spin them down and place on ice.

A	B	C
95 °C for 3min, cool down on ice immediately for at least 5 min		
Reagents	Per 1 sample µL	Per 100 samples µL
PEG-4000 (50%)	10	1000
H2O	8	800
T4 ligase buffer (10X final)	5	500
T4 PNK (10,000 units/mL)	1	100
Add the above reagents one by one and then mix for the 1st time		
Forward adapter (1 pmol)	1	100
Reserve adapter (1 pmol)	1	100
Add the above adapters one by one and then mix for the 2nd time		
T4 DNA ligase (400,000 units/mL)	2	200
Add the above reagents one by one and then by finger flicking to mix		
Final volume	50	50000
37 °C for 60 min (set the lid-heating off)		

Note

CRITICAL STEP for prepare master mix:

Make sure the T4 ligase buffer and PEG are dissolve and mix thoroughly;
Add the needed volume of PEG-4000 (sticky, do it slowly), ligase buffer and PNK first and mix thoroughly, then add adapters and mix again, ligase should be added lastly during cooling down the samples and mix thoroughly without vortexing.

5.10 Add 28 µL ligation master mix to the denatured samples, mix thoroughly with pipette, spin briefly.

5.11 Transfer to ThermoCycle for 01:00:00 ligation at 37 °C . 1h

5.12 **Purified with 1X AMPure XP beads (25 µL / 25 µL PCR reaction) and eluted with 22 µL MiliQ H_2O according to the purification procedures from Step7.**

Note

PAUSE POINT Store in fridge for short-time or freezer until beads clean-up.

CAUTION
Ensure that you have booked a ThermoCycle and preheated it to 95 °C .
Pipette, filtered pipette tips, and workbench need to be sterilized by UV lamp for 00:20:00 prior work.
During preparation, reagents and PCR plate must be on ice/cold block. Reagents are stored at -20 °C .
Spin down reagents before using them. Avoid vortexing enzymes.
Remember to include a Positive (Mock from extraction), Negative (H_2O from extraction) & Blank (H_2O for the ligation) controls for the reaction.

Step6: Index PCR

6 **Checklist before starting**

Note

Where: Seq-lab

Timing: 30 mim

Reagents
AccuPrime^{TM} Taq DNA Polymerase
System: #12339016
Nextera XT Index Kit v2 Sets: #FC-131-2001, 2002, 2003, 2004

Equipment
Thermocycler, Minicentrifuge

Procedure

6.1 Take the index primer (illumina i5 + i7) plate from the freezer to the fridge before the start.

6.2 According to the number of samples (include an extra 3 for pipetting errors), prepare a master mix containing per sample. The table below is calculated for a whole plate of 96 wells (Master Mix per 100 samples):

A	B	C
	**Per 1 sample µL**	**Per 100 samples µL**
Purified ligated DNA	21.1	
10× AccuPrime buffer	2.5	250
AccuPrime DNA polymerase	0.4	40
Primer P5 (i5)	1	
Primer P7 (i7)		
Final volume	25	

6.3 In a clean EP tube, transfer the needed volume of 10× AccuPrime buffer and AccuPrime DNA polymerase from the stock solution, gently mix, and spin down.

6.4 Transfer 2.9 µL Master mix to each sample.

6.5 Transfer 1 µL Primer mix to each sample.

6.6 Carefully mix them and spin them down.

6.7 Carefully place the PCR plate into the thermocycle machine and select the following program:

A	B	C
Temperature profile		
95°C	2min	
95°C	15s	x 20 cycles
57°C	30s	
68°C	30s	
4°C	∞	

6.8 Purified with 0.8X AMPure XP beads (20 µL/25 µL PCR reaction) and eluted with 22 µL MiliQ H2O according to the purification procedures from go to step #7.

Note

PAUSE POINT Store in fridge for short-time or freezer until beads clean-up.

CAUTION
During the preparation of index PCR, reagents and PCR plate must be on ice/cold block.
Lead opening while on cold block. Reagents are stored at -20°C.
Gently mix and spin down reagents before using them. Avoid vortexing enzymes.
WARNIN!!! Change pipette tips and PCR leads during work process!

Step7: AMPure beads clean up and library quality check

27m

7 **Checklist before starting**

Note

Where: Seq-lab

Timing: 30 min

Reagents
AMPure XP beads, 60 mL: #A63881
Qubit 1X dsDNA HS Assay Kit: #Q33231
High Sensitivity D5000
ScreenTape Assay: #5067-5593

Equipments/kits
HulaMixture, Invitrogen™
Qubit™ 4 Fluorometer, TapeSation4200 with high-sensitive D5000 Screen Tape (#5067-5592)

Procedure

7.1 Place AMPure XP beads into the Hula mixer to resuspend the beads and to equilibrate to Room temperature for 00:15:00 . 15m

7.2 Label an Eppendorf tube or PCR plate with your sample ID and transfer the 25 µL PCR product.

7.3 Transfer 20 µL (!!! 0.8X) of Beads solution to each PCR product (25 µL) and mix with 100 µL pipette tips (10 times up and down), resulting in a 45 µL mixture.

7.4 Incubate for 00:05:00 at Room temperature . 5m

7.5 Place the tube or plate with the mixture into the magnetic rack for 2-4 min, until the liquid is clear.

7.6 Carefully remove 40 µL the liquid/supernatant with 100 µL pipette tips and discard it, keep 5 µL without disturbing the beads-pellet.

7.7 Wash the beads-pellet with 175 µL freshly prepared Fresh 80% Ethanol **Contributed by users** by gently dispensing it over the beads with 200 µL pipette tips. Let it rest for 00:00:30 and then remove the liquid. 30s

7.8 Repeat washing go to step #7.7 .

7.9 Spin the tudes or plates in a minicentrifuge for 00:00:30 to collect all the residual liquid. 30s

7.10 Put the tubes or plates on a magnetic rack and remove the excess with 10 µL pipette tips.

7.11 Air dry for approximately 00:00:30 to evaporate the Ethanol. 30s

7.12 Remove the tube or plate from the magnetic rack. Add 16 µL nuclease free water and mix with a pipette (>10 times up and down) to resuspend the beads-pellet. Incubate for 00:02:00 at Room temperature . 2m

7.13 Spin down for 00:00:30 if there is liquid on the wall of tubes or well, place the tube or plate back on the magnetic rack and wait for 00:03:00 until the liquid clears. 3m 30s

7.14 Transfer 15 µL of the liquid/supernatant to a new, labeled Eppendorf tube or a new plate.

7.15 Qubit measurement of cleaned PCR products and pool 10 ng per sample to prepare a pooled library for library quality check with TapeStation following their standard protocols.

Note

PAUSE POINT Send for sequencing or store in the fridge 4 °C for short time or freezer -80 °C for long time.
