# Supplementary material for: A single-stranded based library preparation method for virome characterization
Source: Microbiome. 2024 Oct 24;12:219. doi: 10.1186/s40168-024-01935-5 (PMC11515303; doi:10.1186/s40168-024-01935-5)
Supplement: Supplementary file 3 — Additional file 2. SSLR adapter design. [file 40168_2024_1935_MOESM2_ESM.docx]

Additional file 2: SSLR adapter design

Forward adapter (P5):

/5AmMC6/TCGTCGGCAGCGTCAGATGTGTATAAGAGACAG

/3AmMO/AGCAGCCGTCGCAGTCTACACATATTCTCTGTCNNNNNNN/6CMmA5/

Reverse adapter (P7):

/5Phos/CTGTCTCTTATACACATCTCCGAGCCCACGAGAC/3AmMO/

/3AmMO/NNNNNNNGACAGAGAATATGTGTAGAGGCTCGGGTGCTCTG/5AmMC6/

3AmMO, 5AmMC6: modification and block parts

5Phos: 5’ Phosphate groups

Nucleotides in yellow background are the Illumina Paired-End Adapter Sequences
