# Supplementary material for: A single-stranded based library preparation method for virome characterization
Source: Microbiome. 2024 Oct 24;12:219. doi: 10.1186/s40168-024-01935-5 (PMC11515303; doi:10.1186/s40168-024-01935-5)
Supplement: Supplementary file 4 — Additional file 3. Metavirome sequencing and data pre-processing. [file 40168_2024_1935_MOESM3_ESM.docx]

Additional file 3: Metavirome sequencing and data pre-processing

**Preprocessing of raw sequencing data of mock samples**

The mock metavirome generates a median of 2,483,360 reads with 150 bp paired-end sequencing (range, 165,994 - 12,192,992 reads, **Supplementary Table S3**). All the raw sequencing reads from Nextera XT, MDA_0.5h, MDA_1.5h and SSLR were trimmed from adaptors and barcodes and the high-quality sequences (>95% quality) using Trimmomatic v0.35 [1], with using NexteraPE-PE and a minimum size of 50nt were retained for further analysis. All the adaptors of raw reads from xGen library were removed by Trimmomatic and then the low complexity adaptase tail from first and last 8 nucleotides of each read were trimmed. After trimming, all the reads were subjected to within-sample de-novo assembly-only using metaSpades v3.15.1 [2], and the contigs with a minimum length of 2,200 nt were retained. Contigs generated from all samples were pooled and de-replicated at 90% identity using BBMap tool (dedupe.sh) [3]. Prediction of viral contigs/genomes was carried out using VirSorter2 [4] (“full” categories|dsDNAphage, ssDNA, RNA, Lavidaviridae, nucleocytoplasmic large DNA viruses (NCLDV)|viral quality≥0.66), vibrant [5] (High-quality|Complete), and checkv [6] (High-quality|Complete). Taxonomy of mock community was inferred by blasting viral ORF against a customized phage mock taxonomy database (https://github.com/XC-Zhai/SSLR/tree/main/Customed_db) retrieved from NCBI and the Lowest Common Ancestor (LCA) for every contig was estimated based on a minimum e-value of 10e-5. Following assembly, quality control, and annotations, reads from all samples were mapped against the viral (high-quality) contigs (vOTUs) using the bowtie2 (version 2.2.5, default parameters) [7] and a contingency-table of reads per Kbp of contig sequence per million reads sample (RPKM) was generated, here defined as vOTU-table. Code describing this pipeline can be accessed on github: github.com/jcame/virome_analysis-FOOD. Genome assembly metrics before and after quality check were generated using QUAST (v.4.6.3) with default parameter [8]. The coverage of each genome was generated by converting sam file of Bowtie2 alignment using samtools.

**Estimation of sequencing depths and error rates**

To estimate the sequencing depth of each phage genome from all mocks in the present study, SAM files generated by bowtie2 contigs mapping back to clean reads to BAM format using Samtools (v 1.9) Mapping percentage was calculated during Bowtie2 alignment and visualized by ggplot2.

To test whether heat and DMSO treatment cause any effect on sequencing fidelity during SSLR, we used two approaches to estimate sequencing error rates according to the previous method [9]. R package ShadowRegression (v1.18) was used to calculate the error rate from each treatment, and then evaluated for differences using robust linear regressions [10]. The detailed script can be found from: https://github.com/awilcox83/dsRNA-sequencing.

**Data pre-processing of metavirome of human fecal sequencing**

The fecal metavirome generates a median of 4,681,524 reads with 150 bp paired-end sequencing (range, 2,238,562 - 6,881,604 reads, **Supplementary Table S8**). The raw reads were trimmed as described above and checked for the presence of Phi X174 using BBMap tool (bbduk.sh) before MetaSpades assembly [3]. Random forward reads of 50,000 of each samples were subjected to the Kraken2 program to map human, archaea, bacteria, viral, plasmid, UniVec Core with a standard pre-built reference database for the estimation of the contaminations [11]. All the assembled contigs from each sample were pooled together and quality check with checkv, vibrant and virsorter was carried out as described above to generate high-quality contigs. Following assembly, quality control, and annotations, reads from all samples were mapped against the viral (high-quality) contigs (vOTUs) using the bowtie2 (version 2.2.5, default parameters) [7] and a contingency-table of reads per Kbp of contig sequence per million reads sample (RPKM) was generated, here defined as vOTU-table.

**Fecal virome taxonomy**

Taxonomy of the 1388 high-quality fecal virome contigs was inferred by blasting viral ORF against Virus Orthologous groups (VOG) release 217 (VOG217) database [12], COPSAC infant viruses [13] and human gut archaeal viruses [14] and the Lowest Common Ancestor (LCA) for every contig was estimated based on a minimum e-value of 10e-5.

**Host prediction of 34 representative virome contigs**

The 34 representative contigs were subjected to iPHoP v1.3.2 (integrated Phage Host Prediction) [15] to predict their host at genus level with default parameters by using its “add_to_db” function to add the 145 reference genomes of Sphingomonas species retrieved from NCBI.

**Searching PAU phage from 4 virus databases**

Usearch10 [16] and MMseqs2 [17] were used to search the existence of PAU phage from 4 gut virome databases, namely GVD (gut virome database) [18], GPD (gut phage database) [19], MGV (metagenomic gut virus) [20] and high-confidence IMG/VR4.1 (Integrated Microbial Genomes / Viruses) [21] with e-value of 10e-5. The best hits (top 5) were listed in **Supplementary Table S9**.

**References:**

1. Bolger AM, Lohse M, Usadel B: Trimmomatic: a flexible trimmer for Illumina sequence data. *Bioinformatics* 2014, 30(15):2114-2120.

2. Bankevich A, Nurk S, Antipov D, Gurevich AA, Dvorkin M, Kulikov AS, Lesin VM, Nikolenko SI, Pham S, Prjibelski AD *et al*: SPAdes: A New Genome Assembly Algorithm and Its Applications to Single-Cell Sequencing. *J Comput Biol* 2012, 19(5):455-477.

3. Bushnell B: BBMap: A Fast, Accurate, Splice-Aware Aligner. *Lawrence Berkeley National Laboratory* 2014, LBNL-7065E.

4. Guo JR, Bolduc B, Zayed AA, Varsani A, Dominguez-Huerta G, Delmont TO, Pratama AA, Gazitua MC, Vik D, Sullivan MB *et al*: VirSorter2: a multi-classifier, expert-guided approach to detect diverse DNA and RNA viruses. *Microbiome* 2021, 9(1).

5. Kieft K, Zhou ZC, Anantharaman K: VIBRANT: automated recovery, annotation and curation of microbial viruses, and evaluation of viral community function from genomic sequences. *Microbiome* 2020, 8(1).

6. Nayfach S, Camargo AP, Schulz F, Eloe-Fadrosh E, Roux S, Kyrpides NC: CheckV assesses the quality and completeness of metagenome-assembled viral genomes. *Nat Biotechnol* 2021, 39(5):578-585.

7. Langmead B, Salzberg SL: Fast gapped-read alignment with Bowtie 2. *Nat Methods* 2012, 9(4):357-U354.

8. Gurevich A, Saveliev V, Vyahhi N, Tesler G: QUAST: quality assessment tool for genome assemblies. *Bioinformatics* 2013, 29(8):1072-1075.

9. Wilcox AH, Delwart E, Díaz-Muñoz SL: Next-generation sequencing of dsRNA is greatly improved by treatment with the inexpensive denaturing reagent DMSO. *Microbial genomics* 2019, 5(11):e000315.

10. Wang XV, Blades N, Ding J, Sultana R, Parmigiani G: Estimation of sequencing error rates in short reads. *Bmc Bioinformatics* 2012, 13.

11. Lu J, Rincon N, Wood DE, Breitwieser FP, Pockrandt C, Langmead B, Salzberg SL, Steinegger M: Metagenome analysis using the Kraken software suite. *Nature Protocols* 2022.

12. Grazziotin AL, Koonin EV, Kristensen DM: Prokaryotic Virus Orthologous Groups (pVOGs): a resource for comparative genomics and protein family annotation. *Nucleic Acids Research* 2017, 45(D1):D491-D498.

13. Shah SA, Deng L, Thorsen J, Pedersen AG, Dion MB, Castro-Mejia JL, Silins R, Romme FO, Sausset R, Jessen LE *et al*: Expanding known viral diversity in the healthy infant gut. *Nat Microbiol* 2023, 8(5):986-998.

14. Li R, Wang YM, Hu H, Tan Y, Ma YF: Metagenomic analysis reveals unexplored diversity of archaeal virome in the human gut. *Nat Commun* 2022, 13(1).

15. Roux S, Camargo AP, Coutinho FH, Dabdoub SM, Dutilh BE, Nayfach S, Tritt A: iPHoP: An integrated machine learning framework to maximize host prediction for metagenome-derived viruses of archaea and bacteria. *Plos Biol* 2023, 21(4).

16. Edgar RC: Search and clustering orders of magnitude faster than BLAST. *Bioinformatics* 2010, 26(19):2460-2461.

17. Steinegger M, Soding J: MMseqs2 enables sensitive protein sequence searching for the analysis of massive data sets. *Nat Biotechnol* 2017, 35(11):1026-1028.

18. Gregory AC, Zablocki O, Zayed AA, Howell A, Bolduc B, Sullivan MB: The Gut Virome Database Reveals Age-Dependent Patterns of Virome Diversity in the Human Gut. *Cell Host Microbe* 2020, 28(5):724-740 e728.

19. Camarillo-Guerrero LF, Almeida A, Rangel-Pineros G, Finn RD, Lawley TD: Massive expansion of human gut bacteriophage diversity. *Cell* 2021, 184(4):1098-1109 e1099.

20. Nayfach S, Paez-Espino D, Call L, Low SJ, Sberro H, Ivanova NN, Proal AD, Fischbach MA, Bhatt AS, Hugenholtz P *et al*: Metagenomic compendium of 189,680 DNA viruses from the human gut microbiome. *Nat Microbiol* 2021, 6(7):960-970.

21. Camargo AP, Nayfach S, Chen IMA, Palaniappan K, Ratner A, Chu K, Ritter SJ, Reddy TBK, Mukherjee S, Schulz F *et al*: IMG/VR v4: an expanded database of uncultivated virus genomes within a framework of extensive functional, taxonomic, and ecological metadata. *Nucleic Acids Research* 2023, 51(D1):D733-D743.
