# Supplementary material for: A single-stranded based library preparation method for virome characterization
Source: Microbiome. 2024 Oct 24;12:219. doi: 10.1186/s40168-024-01935-5 (PMC11515303; doi:10.1186/s40168-024-01935-5)
Supplement: Supplementary file 5 — Additional file 4. Supplementary Table S1-S11. TableS1: Summary of DNA genome sequences. Top table: Relative abundance and coverage of phage genomes in libraries from DNA mock communities (Mock A, Mock B and Mock C) with T4 genome. The relative abundance of each genome was calculated based on its coverage by using bowtie2 alignment with our customed database (https://github.com/XC-Zhai/SSLR/tree/main/Customed_db). For comparing the efficiency of different methods for each group of phages (i.e., dsDNA and ssDNA), relative abundance of two type genomes were computed from this table (by dividing the value for each phage by the sum for the group). Middle table: The average coverage for each genome from each library preparation method. Bottom table: similar to top table without T4 genome. TableS2: Comparisons of cost and effectiveness of Mock virome library preparation method. This comparison only considers the library preparation step for DNA virome. TableS3: Overview of metavirome sequencing for the DNA phage mock community. Statistics from assembly before and after quality checks with CheckV, vibrant and virsorter2. Contigs from each library prepared methods were evaluated by quality checks with CheckV, vibrant and virsorter2 and then subjected to the Quast for quality assessment of assembled contigs. The theatrical statistics of mock community were also subjected to Quast and bolded at the bottom of table (in yellow background). TableS4: Summary of contigs hits to the customed mock database. The quality-checked contigs were subjected to the customed database and the hit number was counted and listed in the table. TableS5: Summary of DNA genome sequences with interested modified genomes (T4 and T4-c). T4 is a highly modified genome, T4-c has lower modification compared to T4 genome. TableS6: Summary of DNA/RNA genome sequences prepared with SSLR method. Relative abundance and coverage of phage genomes in libraries from DNA/RNA mock communities (Mock D, Mock E and Mock [file 40168_2024_1935_MOESM4_ESM.pdf]

Legends

Table S1: Summary of DNA genome sequences. Top table: Relative abundance and coverage of phage genomes in libraries from DNA mock communities (Mock A, Mock B and Mock C) with T4 genome. The relative abundance of each genome was calculated based on its coverage by using bowtie2 alignment with our customed database (https://github.com/XC-Zhai/SSLR/tree/main/Customed_db). For comparing the efficiency of different methods for each group of phages (i.e., dsDNA and ssDNA), relative abundance of two type genomes were computed from this table (by dividing the value for each phage by the sum for the group). Middle table: The average coverage for each genome from each library preparation method. Bottom table: similar to top table without T4 genome.

Table S2: Comparisons of cost and effectiveness of Mock virome library preparation method. This comparison only considers the library preparation step for DNA virome.

Table S3: Overview of metavirome sequencing for the DNA phage mock community. Statistics from assembly before and after quality checks with CheckV, vibrant and virsorter2. Contigs from each library prepared methods were evaluated by quality checks with CheckV, Vibrant and Virsorter2 and then subjected to the Quast for quality assessment of assembled contigs. The theatrical statistics of mock community was also subjected to Quast and bolded at the bottom of table (in yellow backgroud).

Table S4: Summary of contigs hits to the customed mock database. The quality-checked contigs were subjected to the customed database and the hit number was counted and listed in the table.

Table S5: Summary of DNA genome sequences with interested modified genomes (T4 and T4-c). T4 is a highly modified genome, T4-c is has lower modification compared to T4 genome.

Table S6: Summary of DNA/RNA genome sequences prepared with SSLR method. Relative abundance and coverage of phage genomes in libraries from DNA/RNA mock communities (Mock D, Mock E and Mock F) with different treatments (heat, no heat, DMSO and no DMSO). The relative abundance of each genome was calculated based on its coverage by using bowtie2 alignment with our customed database. For comparing the efficiency of different treatments for each type of phage genome (DNA and RNA), relative abundance of two type genomes (DNA or RNA) were calculated from this table (by dividing the value for each phage by the sum for the genome types). Middle table: The average coverage for each genome from each library preparation methods. The average coverage for each genome from each treatment method is listed in the bottom table.

Table S7: Taxonomic classification of fecal metavirome sequencing reads into the categories viral, human, bacterial, and unknown origin. To check the presence of non-viral DNA sequences, 50,000 random forward reads were used according to their match to a range of viral, bacterial, and human reference database of Kaiju 1.8.2.

Table S8: Relative abundance of fecal virome prepared with 5 different library strategies at taxonomy of family level (support data for **Fig.5B**) and the overall alignment rate of different library preparation methods based on bowtie2 alignment to the vog217 database.

Table S9: Top 5 hits of PAU phage on 4 virus databases (GVD, GPD, MGV and IMG_VR4.1).

Table S10: Characteristics of phage genomes included in the mock communities from the present study, as well as growth conditions for the strains in the mock communities.

Table S11: Averagae alignment rate of each library to the customed databases for all the mock communities tested in the present study.

Table S1

Relative abundance(%) with T4

Genome type	Phage	Mock A Community MA (input)	MDA_0.5h_MA	MDA_1.5h_MA	Nextera_MA	SSLR_MA	xGen_MA	Mock B Community MB (input)	VIDA_0.5h_ME	MDA_1.5h_MB	Nextera_MB	SSLR_MB	xGen_MB	Mock C Community MC (input)	MDA_0.5h_MC	MDA_1.5h_MC	Nextera_MC	SSLR_MC	xGen_MC
Total reads	--	--	5226080	5048554	3194866	5380278	242612	--	4980678	4490090	1152164	4667526	232148	--	4993056	4510124	456606	12192992	269190
dsDNA	phi29	**13.04**	9.32	8.67	11.91	10.37	13.04	7.69	4.91	4.55	12.57	7.40	8.09	1.64	1.43	1.16	13.77	1.82	2.66
dsDNA	T7	**13.04**	13.12	11.78	24.52	11.42	12.69	7.69	7.50	6.30	23.00	7.52	8.43	1.64	1.79	1.37	20.86	1.63	2.54
dsDNA	c2	**13.04**	4.77	4.12	8.72	12.22	11.88	7.69	2.28	1.88	9.10	6.43	7.09	1.64	0.66	0.45	11.64	1.59	2.91
dsDNA	lambda	**13.04**	15.79	15.31	15.29	10.38	10.34	7.69	7.78	7.57	14.56	7.75	6.88	1.64	1.92	1.65	12.32	1.33	2.12
dsDNA	P35	**13.04**	14.50	14.72	15.79	12.59	12.93	7.69	7.71	7.78	16.32	7.00	8.15	1.64	1.83	1.61	15.51	1.80	2.26
dsDNA	P1	**13.04**	14.27	12.83	20.52	12.84	11.40	7.69	6.92	5.78	20.25	8.10	7.91	1.64	1.63	1.25	19.51	1.05	2.79
dsDNA	T4	**13.04**	0.22	0.09	3.00	26.10	23.18	7.69	0.13	0.05	2.86	12.46	27.46	1.64	0.02	0.01	2.78	5.44	6.02
ssDNA	phi-X174	**4.35**	17.13	20.17	0.10	1.94	2.15	23.08	39.87	42.56	0.54	20.96	13.27	44.26	58.05	58.89	1.16	41.68	38.07
ssDNA	M13mp18	**4.35**	10.88	12.30	0.15	2.14	2.39	23.08	22.90	23.53	0.80	22.39	12.72	44.26	32.67	33.60	2.45	43.66	40.64
**All dsDNA**	--	**91.30**	**71.98**	**67.52**	**99.75**	**95.92**	**95.46**	**53.85**	**37.23**	**33.91**	**98.66**	**56.66**	**74.01**	**11.48**	**9.28**	**7.50**	**96.40**	**14.66**	**21.29**
**All ssDNA**	--	**8.70**	**28.02**	**32.48**	**0.25**	**4.08**	**4.54**	**46.15**	**62.77**	**66.09**	**1.34**	**43.34**	**25.99**	**88.52**	**90.72**	**92.50**	**3.60**	**85.34**	**78.71**
**ssDNA proportion**		**--**	**3.22**	**3.73**	**34.99**	**2.13**	**1.91**	**--**	**1.36**	**1.43**	**34.45**	**0.94**	**0.56**	**--**	**1.02**	**1.04**	**24.56**	**0.96**	**0.89**
**T4 proportion variation to**		**--**	**60.55**	**148.04**	**4.34**	**2.00**	**1.78**	**--**	**61.29**	**140.72**	**2.69**	**1.62**	**3.57**	**--**	**86.96**	**159.82**	**1.70**	**3.32**	**3.67**

MeanDepth

Genome type	Phage	Mock A Community MDA_0.5h_MA	MDA_1.5h_MA	Nextera_MA	SSLR_MA	xGen_MA	Mock B Community VIDA_0.5h_ME	VIDA_1.5h_ME	Nextera_MB	SSLR_MB	xGen_MB	Mock C Community MDA_0.5h_MC	MDA_1.5h_MC	Nextera_MC	SSLR_MC	xGen_MC
dsDNA	phi29	482.37	424.45	479.71	1103.36	217.40	185.91	142.31	280.75	585.98	121.40	39.52	28.32	157.77	281.92	34.66
dsDNA	T7	328.75	279.06	477.06	587.17	102.03	137.29	95.36	248.03	325.72	61.13	23.89	16.04	115.46	126.68	16.02
dsDNA	c2	214.91	175.55	305.32	1130.80	172.54	75.07	51.33	176.72	580.25	92.45	15.93	9.54	116.06	311.73	33.08
dsDNA	lambda	325.56	298.27	245.02	439.62	68.76	117.12	94.25	129.41	243.78	41.28	21.19	15.96	56.19	92.42	11.00
dsDNA	P35	404.44	387.77	342.59	721.74	116.06	157.11	131.10	196.23	383.90	65.84	27.24	21.10	95.72	150.73	15.92
ssDNA	M13mp18	1495.15	1597.54	16.21	606.15	106.84	2292.91	1947.36	47.27	2181.65	511.24	2386.43	2155.77	74.13	9425.07	1437.31
dsDNA	P1	150.84	128.19	168.43	278.40	38.62	53.39	36.85	92.16	146.77	24.13	9.19	6.18	45.55	61.95	7.38
ssDNA	phi-X174	3168.89	3526.49	13.66	739.49	128.96	5374.06	4740.97	42.38	3096.08	718.57	5710.36	5087.09	46.70	13754.90	1814.22
dsDNA	T4	1.28	0.49	13.85	317.52	44.77	0.54	0.20	7.32	257.31	48.04	0.06	0.03	3.65	96.28	9.16

* Meandepth was calculated from bowtie2 alignment

Relative abundance (%) without T4

Genome type	Phage	Mock A Community MA (input)	MDA_0.5h_MA	MDA_1.5h_MA	Nextera_MA	SSLR_MA	xGen_MA	Mock B Community MB (input)	VIDA_0.5h_ME	MDA_1.5h_MB	Nextera_MB	SSLR_MB	xGen_MB	Mock C Community MC (input)	MDA_0.5h_MC	MDA_1.5h_MC	Nextera_MC	SSLR_MC	xGen_MC
Total reads	--	--	5169314	4489158	3501228	4523574	275346	--	4602904	3939584	1017930	8993604	165994	--	4781552	4308190	437006	11666060	174058
dsDNA	phi29	**15.00**	9.38	8.96	12.24	14.18	16.44	8.33	4.89	4.77	12.53	9.95	11.50	1.67	1.44	1.19	14.57	3.10	2.84
dsDNA	T7	**15.00**	12.79	11.84	24.98	15.16	17.13	8.33	7.56	6.86	23.95	11.31	12.11	1.67	1.84	1.44	20.14	2.83	2.66
dsDNA	c2	**15.00**	4.82	4.12	9.05	17.17	15.16	8.33	2.26	1.90	9.23	11.11	9.93	1.67	0.49	0.35	13.74	3.85	2.83
dsDNA	lambda	**15.00**	15.80	14.56	15.64	13.95	14.51	8.33	8.28	7.54	14.88	10.34	9.79	1.67	1.94	1.77	11.89	2.53	2.41
dsDNA	P35	**15.00**	14.91	14.12	16.42	17.60	16.29	8.33	7.96	7.68	16.87	12.42	11.47	1.67	1.88	1.63	15.37	2.95	2.29
ssDNA	M13mp18	**5.00**	10.97	12.37	0.16	2.83	3.10	25.00	22.85	22.04	0.89	15.04	16.70	45.00	33.91	33.66	2.83	39.25	42.87
dsDNA	P1	**15.00**	14.07	12.23	21.40	16.54	14.59	8.33	6.92	6.07	21.05	12.74	10.90	1.67	1.59	1.22	20.24	3.28	2.76
ssDNA	phi-X174	**5.00**	17.26	21.80	0.11	2.58	2.78	25.00	39.29	43.13	0.61	17.11	17.61	45.00	56.91	58.75	1.23	42.20	41.33
**All dsDNA**	--	**90.00**	**71.77**	**65.83**	**99.73**	**94.60**	**94.12**	**50.00**	**37.87**	**34.82**	**98.51**	**67.87**	**65.70**	**10.00**	**9.18**	**7.60**	**95.95**	**18.54**	**15.80**
**All ssDNA**	--	**10.00**	**28.23**	**34.17**	**0.27**	**5.41**	**5.88**	**50.00**	**62.14**	**65.17**	**1.50**	**32.15**	**34.30**	**90.00**	**90.82**	**92.41**	**4.06**	**81.45**	**84.20**
**ssDNA proportion variation to expected**		**--**	**2.82**	**3.42**	**37.04**	**0.54**	**0.59**	**--**	**1.24**	**1.30**	**33.33**	**0.64**	**0.69**	**--**	**1.01**	**1.03**	**22.17**	**0.91**	**0.94**

Table S2

Library method	Template input	Hands on time	Walk-way time	Number of purifications[#]	Cost	Mock Capturability
Nextera XT	1ng/10ul	30min	60min	1	~ $32	7/9
MDA_0.5h	10ng/10ul	65min	115min	2	~ $38	8/9
MDA_1.5h	10ng/10ul	65min	175min	2	~ $38	8/9
**SSLR**	**10ng/20ul**	**30min**	**135min**	2~3	~ **$16**	**9/9**
xGen (1s plus)	10 pg–250 ng/20ul	40min	150min	3~4	~ $42	9/9

* This comparison only considers the library preparation step for DNA virome.

# Number of Total purification times (including Beads or kit purification).

Table S3

Biome	Library	Sample	T4	Reads: #Raw reads	# Clean reads	% reads passed QC	Bowtie2 reads	% Bowtie2 mapping rate	% Bowtie2 mapping rate to raw reads	Mapped reads	Mapping rate to raw reads	Contigs after Assembly: Assembled Contigs	GC%	N50	N75	L50	L75	N's per 100 kbp	Largest contig	Contigs after QC: Assembled Contigs	GC%	N50	N75	L50	L75	N's per 100 kbp	Largest contig
		A1	-	5169314	1478000	28.59	373852	25.29	7.23	656432	12.70	14	46.06	39878	18898	3	6	0	79009	11	45.89	48259	16357	2	5	0	79009
		A2	+	5226080	1497277	28.65	382638	25.56	7.32	668033	12.78	18	46.04	20032	16357	4	8	0	78502	13	46.32	36267	16357	2	5	0	78502
	MDA_0.5h	B1	-	4602904	674900	14.66	250967	37.19	5.45	448259	9.74	13	45.46	25268	19129	4	7	3.71	47449	12	45.9	39879	19129	3	6	4	47449
		B2	+	4980678	717659	14.41	274781	38.29	5.52	486851	9.77	13	45.7	47464	16357	2	5	0	89447	10	46.1	47464	18770	2	4	0	89447
		G1	-	4781552	417551	8.73	184744	44.24	3.86	326864	6.84	20	46.03	16357	10756	5	9	8.29	48032	13	47.05	18538	16357	3	5	0	48032
		G2	+	4993056	437517	8.76	203605	46.54	4.08	355391	7.12	28	45.61	14680	7405	6	12	0	28472	14	46.98	21014	7821	3	6	0	28472
		A3	-	4489158	1172226	26.11	329964	28.15	7.35	584699	13.02	14	46.11	39833	20304	4	6	0	48259	9	46.22	43672	39833	3	4	0	48259
		A4	+	5048554	1320361	26.15	356025	26.96	7.05	631065	12.50	15	46.05	39833	16357	3	6	0	78502	10	46.44	48291	18688	2	4	0	78502
	MDA_1.5h	B3	-	3939584	541865	13.75	329964	60.89	8.38	401147	10.18	13	45.56	37862	19285	4	6	0	51640	8	46.37	39767	19570	2	4	0	51640
		B4	+	4490090	586951	13.07	223739	38.12	4.98	402471	8.96	12	45.44	37070	19127	4	6	3.72	48427	10	46.71	37571	37070	3	4	4.57	48427
		G3	-	4308190	374427	8.69	169172	45.18	3.93	301278	6.99	28	46.35	10472	6432	7	13	0	22990	17	47.17	16629	8395	4	8	0	22990
		G4	+	4510124	382606	8.48	175421	45.85	3.89	311976	6.92	30	45.74	10563	5463	6	14	4.49	39294	16	47.17	16328	5463	3	7	0	39294
		A5	-	3501228	1416715	40.46	287872	20.32	8.22	535093	15.28	12	46.31	39833	19285	3	5	0	89337	7	46	48504	22204	2	4	0	89337
		A6	+	3194866	1346231	42.14	281341	20.90	8.81	518313	16.22	35	42.58	20180	11176	5	12	0	89337	31	41.97	22216	9109	4	11	0	89337
Mock community	Nextera	B5	-	1017930	529658	52.03	287872	54.35	28.28	259080	25.45	9	45.61	48490	22216	2	4	0	89337	7	45.99	48490	22216	2	4	0	89337
		B6	+	1152164	598415	51.94	152493	25.48	13.24	286979	24.91	33	43.33	22156	7870	4	9	0	89337	26	43.56	39833	9103	3	7	0	89337
		G5	-	437006	244924	56.05	73105	29.85	16.73	137759	31.52	8	45.52	48463	22216	2	4	0	89337	7	45.99	48463	22216	2	4	0	89337
		G6	+	456606	261991	57.38	78120	29.82	17.11	147126	32.22	16	44.81	39833	19285	3	5	0	89337	13	45.35	48451	22173	2	4	0	89337
		A7	-	4523574	1477226	32.66	467699	31.66	10.34	870316	19.24	13	46.01	39833	19285	3	6	0	89447	9	45.85	48575	22225	2	4	0	89447
		A8	+	5380278	2395993	44.53	745545	31.12	13.86	1370982	25.48	16	42.38	89337	23790	2	5	0	168110	10	41.62	89337	39833	2	4	0	168110
	SSLR	B7	-	8993604	1854108	20.62	620104	33.44	6.89	1133956	12.61	16	46	39833	19285	3	6	0	89337	9	45.86	48554	22311	2	4	0	89337
		B8	+	4667526	1522107	32.61	557564	36.63	11.95	1018370	21.82	18	42.11	79864	22216	2	5	0	168107	10	41.47	79864	39833	2	4	0	168107
		G7	-	11666060	1536974	13.17	567630	36.93	4.87	995106	8.53	12	45.49	32029	19285	3	6	0	89337	9	45.86	39833	22216	2	4	0	89337
		G8	+	12192992	1829854	15.01	716751	39.17	5.88	1284022	10.53	12	41.6	89337	39833	2	4	0	168108	10	41.62	89337	39833	2	4	0	168108
		S1	-	275346	247057	89.73	110003	44.53	39.95	206784	75.10	10	45.41	48490	19285	2	5	0	89337	9	45.85	48490	22216	2	4	0	89337
		S2	-	165994	122576	73.84	71329	58.19	42.97	132425	79.78	10	45.41	48523	19328	2	5	0	89337	9	45.85	48523	22216	2	4	0	89337
	xGen	S3	-	174058	84467	48.53	58620	69.40	33.68	109255	62.77	26	43.88	11894	5419	5	12	0	24335	17	44.23	11894	5441	4	8	0	22217
		S4	+	242612	226980	93.56	111717	49.22	46.05	211350	87.11	11	41.54	89337	39833	2	4	0	168106	10	41.63	89337	39833	2	4	0	168106
		S5	+	232148	184420	79.44	101369	54.97	43.67	189816	81.77	12	41.54	89337	37725	2	4	4.55	168106	11	41.62	89337	37725	2	4	4.76	168106
		S6	+	269190	127168	47.24	91407	71.88	33.96	164531	61.12	35	41.41	19314	9618	7	15	16.64	58307	28	40.9	19314	9690	6	12	16.68	58307
																			**Mock with T4**	**9**	**41.59**	**94800**	**39937**	**2**	**4**	**0.68**	**168903**
																			**Mock without T4**	8	45.48	48502	35822	2	4	0	94800

				Contigs after Assembly: Assembled Contigs	GC%	N50	N75	L50	L75	N's per 100 kbp	Largest contig	Contigs after QC: Assembled Contigs	GC%	N50	N75	L50	L75	N's per 100 kbp	Largest contig
		A1	-	24	45.82	13162	9670	8	13	0	26368	12	47.4	13559	11158	4	7	0	26368
		A2	+	23	45.81	13666	8395	6	11	0	39725	15	47.41	18206	8395	3	7	0	39725
	MDA_0.5h	B1	-	23	45.98	5816	4121	7	13	0	18887	18	47.5	6971	3701	5	11	0	18887
		B2	+	25	45.86	7646	4261	8	14	0	13450	18	47.63	7646	4261	6	10	0	10020
		G1	-	1	42.4	7304	7304	1	1	0	7304	1	42.4	7304	7304	1	1	0	7304
		G2	+	1	40.75	5750	5750	1	1	0	5750	1	40.75	5750	5750	1	1	0	5750
		A3	-	23	45.8	11963	8115	6	11	0	31527	11	47.87	19078	8912	3	5	0	31527
		A4	+	23	45.81	14211	8019	4	11	0	46507	12	47.25	39491	10418	2	5	0	46507
	MDA_1.5h	B3	-	20	46.95	4937	4276	8	13	9.82	9621	15	47.03	5954	4547	6	10	0	9621
		B4	+	21	45.62	5405	3408	7	13	0	13881	14	47.14	5405	4736	6	9	0	9776
		G3	-	1	42.4	7304	7304	1	1	0	7304	1	42.4	7304	7304	1	1	0	7304
		G4	+	1	43.35	8450	8450	1	1	0	8450	1	43.35	8450	8450	1	1	0	8450
		A5	-	11	45.52	24953	23447	4	6	3.88	47690	7	46.3	39833	22121	2	4	0	47690
		A6	+	15	45.61	23005	16750	5	8	0	39833	9	45.8	23005	19274	3	5	0	39833
Mock community	Nextera	B5	-	9	45.51	39833	30489	3	5	0	48467	9	45.51	39833	30489	3	5	0	48467
		B6	+	9	45.54	39833	19285	3	5	0	78430	8	46.01	48404	22128	2	4	0	78430
		G5	-	9	45.52	39833	22216	3	5	0	65453	7	45.63	48460	22216	2	4	0	65453
		G6	+	7	45.51	48441	35769	2	4	0	89337	6	46.26	48441	39833	2	3	0	89337
		A7	-	9	45.43	48515	22301	2	4	0	89337	8	45.88	48515	22301	2	4	0	89337
		A8	+	11	41.5	81642	39833	2	4	0	168106	10	41.59	81642	39833	2	4	0	168106
	SSLR	B7	-	10	45.43	39833	19933	3	5	3.77	69530	8	45.53	48514	22175	2	4	4.42	69530
		B8	+	23	40.18	39789	19285	2	5	2.7	164005	13	39.35	164005	35952	1	3	3.19	164005
		G7	-	10	38.35	4020	3369	4	7	0	7304	7	38.94	3539	3369	3	5	0	7304
		G8	+	7	38.61	5842	4836	3	5	0	7304	6	38.48	5159	4836	3	4	0	7304
		S1	-	9	45.44	48578	22216	2	4	0	89337	8	45.89	48578	22216	2	4	0	89337
		S2	-	9	45.43	48523	22216	2	4	0	89337	8	45.88	48523	22216	2	4	0	89337
	xGen	S3	-	24	43.72	9174	5765	5	11	0	24335	15	44.2	9174	6679	4	8	0	22223
		S4	+	10	41.5	89337	39833	2	4	0	168108	9	41.59	89337	39833	2	4	0	168108
		S5	+	11	41.51	89337	37725	2	4	0	168106	10	41.59	89337	37725	2	4	0	168106
		S6	+	47	41.78	8224	3718	10	23	17.18	26333	34	41.14	10978	4956	6	15	12.96	26333

Table S4

						Mock A					Mock B					Mock C		
				Nextera	MDA_0.5h	MDA_1.5h	SSLR	xGen	Nextera	MDA_0.5h	MDA_1.5h	SSLR	xGen	Nextera	MDA_0.5h	MDA_1.5h	SSLR	xGen
Phage	accession	Genome type	Genome length (bp)	# contigs	# contigs	# contigs	# contigs	# contigs	# contigs	# contigs	# contigs	# contigs	# contigs	# contigs	# contigs	# contigs	# contigs	# contigs
C2	NC001706.1	dsDNA	22 172	1	2	1	1	1	1	2	1	1	1	1	1	3	1	1
T4	NC000866.4	dsDNA	168 903	**22**	**0**	**0**	**1**	**1**	**19**	**0**	**0**	**1**	**1**	**6**	**0**	**0**	**1**	**10**
Phi29	NC011048.1	dsDNA	19 282	1	1	1	1	1	1	1	1	1	1	1	0	1	1	1
P1	NC005856.1	dsDNA	94 800	**2**	**3**	**3**	**2**	**2**	**2**	**2**	**4**	**2**	**2**	**2**	**7**	**6**	**2**	**8**
T7	V01146.1	dsDNA	39 937	1	2	1	1	1	1	1	1	1	1	1	2	1	1	1
P35	DQ003641.1	dsDNA	35 822	2	1	1	1	1	1	1	0	1	1	1	1	1	1	1
Lambda	J02459.1	dsDNA	48 502	1	1	1	1	1	1	1	1	1	1	1	1	2	1	4
Phi X174	NC001422.1	ssDNA (>85%)	5 386	0	1	1	1	1	0	1	1	1	1	0	1	1	1	1
M13mp18	M13mp18	ssDNA	7 249	0	2	1	1	1	0	1	1	1	1	0	1	1	1	1

Table S5

Relative abundance(%)

Genome type	Phage	Mock G Community-T4: MG (input)	MDA_0.5h_MG	MDA_1.5h_MG	Nextera_MG	SSLR_MG	xGen_MG	Mock H Community-T4-c: MH (input)	MDA_0.5h_MH	MDA_1.5h_MH	Nextera_MH	SSLR_MH	xGen_MH
Total raw reads	--	--	1438557	1307872	1768676	7741338	3094046	--	1972868	1870033	1868920	1580348	2784548
dsDNA	phi29	**11.11**	7.68	7.03	10.89	8.34	10.09	**11.11**	6.94	6.62	9.27	6.29	7.18
dsDNA	T7	**11.11**	2.00	1.50	26.19	12.56	14.36	**11.11**	2.04	1.87	26.76	11.43	13.04
dsDNA	c2	**11.11**	3.77	2.72	8.37	7.15	10.56	**11.11**	3.79	2.96	7.84	8.04	8.47
dsDNA	lambda	**11.11**	13.55	12.03	21.70	9.99	19.54	**11.11**	13.71	13.41	22.42	14.76	16.70
dsDNA	P35	**11.11**	8.82	8.66	11.51	16.37	8.94	**11.11**	8.58	8.70	11.07	5.81	7.16
dsDNA	P1	**11.11**	10.66	8.97	19.29	14.95	15.54	**11.11**	9.32	9.15	17.88	11.39	11.71
dsDNA	T4/T4-c	**11.11**	0.19	0.15	1.75	18.57	11.19	**11.11**	0.75	0.50	4.47	31.40	27.78
ssDNA	phi-X174	**11.11**	6.09	7.82	0.13	5.18	4.31	**11.11**	6.44	8.06	0.13	4.70	3.54
ssDNA	M13mp18	**11.11**	47.24	51.13	0.18	6.90	5.46	**11.11**	48.44	48.72	0.17	6.18	4.42
**All dsDNA**	--	**77.78**	**46.67**	**41.05**	**99.70**	**87.92**	**90.23**	**77.78**	**45.12**	**43.22**	**99.70**	**89.12**	**92.04**
**All ssDNA**	--	**22.22**	**53.33**	**58.95**	**0.30**	**12.08**	**9.77**	**22.22**	**54.88**	**56.78**	**0.30**	**10.88**	**7.96**
**ssDNA proportion variation to expected**		**--**	**2.40**	**2.65**	**73.20**	**1.84**	**2.27**	**--**	**2.47**	**2.56**	**73.77**	**0.49**	**0.36**
**T4 proportion variation to expected**		**--**	**59.29**	**74.88**	**6.33**	**1.67**	**1.01**	**--**	**14.86**	**22.01**	**2.49**	**2.83**	**2.50**

MeanDepth

	Mock G Community-T4: MDA_0.5h_MG	MDA_1.5h_M	Nextera_MG	SSLR_MG	xGen_MG	Mock G Community-T4-c: MDA_0.5h_MG	MDA_1.5h_MG	Nextera_MG	SSLR_MG	xGen_MG
Phi29	175.72	218.77	265.51	418.25	713.39	177.43	177.85	262.97	425.33	576.43
T7	39.58	37.00	316.12	325.58	533.35	46.50	44.29	360.69	369.80	502.86
C2	92.84	91.33	190.48	448.75	722.96	100.46	87.53	204.04	472.89	626.71
Lambda	145.75	174.26	252.51	348.88	605.68	164.66	166.01	284.76	390.57	546.10
P35	203.60	263.27	274.14	422.14	705.59	223.88	223.33	300.00	450.81	610.64
P1	75.79	82.19	144.30	221.70	385.01	77.63	77.81	147.44	229.71	323.00
T4/T4-c	0.89	0.84	9.19	132.48	210.90	3.92	2.84	26.24	283.99	356.94
Phi X174	441.02	756.82	27.58	657.65	1010.14	528.96	645.99	29.16	765.20	866.35
M13mp18	2066.51	3065.97	24.00	684.86	885.92	2550.07	2542.34	24.50	787.02	752.31

* Meandepth was calculated from bowtie2 alignment

Table S6

Relative abundance(%)

Genome type	Phage		Mock D Community					Mock E Community					Mock F Community			
		MD (input)	DMSO	No DMSO	Heat	No Heat	ME (input)	DMSO	No DMSO	Heat	No Heat	MF (input)	DMSO	No DMSO	Heat	No Heat
Total reads	--	--	3492348	2843515	3535370	2762825	--	255247	2892220	3082011	3392903	--	3126552	2973092	1326738	3982777
dsDNA	lambda	45.00	48.65	61.27	55.73	75.86	25.00	22.88	33.72	39.96	42.66	5.00	7.54	10.70	12.84	20.90
ssDNA	M13mp18	45.00	25.55	14.25	24.29	10.61	25.00	8.40	9.79	6.72	5.82	5.00	5.03	5.46	4.29	7.12
ssRNA	MS2	5.00	23.25	21.56	17.80	11.62	25.00	56.30	48.43	40.14	40.85	45.00	80.27	75.13	63.75	61.16
dsRNA	Phi6-L	1.67	0.56	0.68	0.63	0.41	8.33	3.02	1.81	3.41	2.40	15.00	2.34	2.04	5.04	2.64
dsRNA	Phi6-M	1.67	1.14	1.46	0.90	0.85	8.33	5.55	3.46	5.48	4.68	15.00	3.00	3.42	7.98	4.60
dsRNA	Phi6-S	1.67	0.85	0.78	0.65	0.66	8.33	3.84	2.79	4.30	3.59	15.00	1.81	3.25	6.10	3.59
**All DNA**	--	**90.00**	**74.20**	**75.52**	**80.03**	**86.47**	**50.00**	**31.28**	**43.51**	**46.67**	**48.48**	**10.00**	**12.57**	**16.16**	**17.13**	**28.02**
**All RNA**	--	**10.01**	**25.80**	**24.48**	**19.97**	**13.53**	**49.99**	**68.72**	**56.49**	**53.33**	**51.52**	**90.00**	**87.43**	**83.84**	**82.87**	**71.98**
**RNA proportion variation to expected**		**--**	**2.58**	**2.45**	**2.00**	**1.35**	**--**	**1.37**	**1.13**	**1.07**	**1.03**	**--**	**0.97**	**0.93**	**0.92**	**0.80**
**DNA proportion variation to expected**		**--**	**0.82**	**0.84**	**0.89**	**0.96**	**--**	**0.63**	**0.87**	**0.93**	**0.97**	**--**	**1.26**	**1.62**	**1.71**	**2.80**

Meandepth

Genome type	Phage	Mock D Community				Mock E Community				Mock F Community			
		DMSO	No DMSO	Heat	No Heat	DMSO	No DMSO	Heat	No Heat	DMSO	No DMSO	Heat	No Heat
dsDNA	lambda	5.93	5.01	182.71	47.50	0.82	1.93	17.40	53.64	1.18	0.52	3.33	36.09
ssDNA	M13mp18	28.00	8.22	401.08	82.85	3.20	3.76	40.16	34.11	6.69	2.04	14.22	49.79
ssRNA	MS2	49.50	24.03	767.92	156.80	42.07	47.31	468.05	1002.97	195.87	72.91	667.40	3030.18
dsRNA	Phi6-L	0.23	0.28	16.35	0.97	0.56	0.37	11.77	12.15	3.02	0.24	14.96	43.97
dsRNA	Phi6-M	1.05	0.87	35.48	4.17	1.54	1.14	35.52	58.53	6.28	0.75	37.84	131.09
dsRNA	Phi6-S	1.28	0.71	38.32	5.43	2.24	1.58	40.69	69.51	6.75	1.34	46.09	162.05

* Meandepth was calculated from bowtie2 alignment

Table S7

Library.Method	Sample	#Reads Eztaxon.16S	#Reads human	#Reads refseq.fungi.bacter	#Reads virus.refseq	#Reads Unclassified	#Reads ITS.unite	% Eztaxon.16S	% human	% refseq.fungi.bacter	% virus.refseq	% Unclassified	% ITS.unite
MDA_0.5h	S1	2195	1507	17670	2886	25742	0	4.39	3.014	35.34	5.772	51.484	0
	S2	2157	1550	17815	2978	25500	0	4.314	3.1	35.63	5.956	51	0
	S3	2412	1496	17141	3011	25940	0	4.824	2.992	34.282	6.022	51.88	0
MDA_1.5h	S1	955	1582	20332	2956	24175	0	1.91	3.164	40.664	5.912	48.35	0
	S2	462	1639	20169	2992	24738	0	0.924	3.278	40.338	5.984	49.476	0
	S3	357	1739	19831	3132	24941	0	0.714	3.478	39.662	6.264	49.882	0
Nextera	S1	1270	1188	28586	730	18226	0	2.54	2.376	57.172	1.46	36.452	0
	S2	1289	1164	28387	694	18466	0	2.578	2.328	56.774	1.388	36.932	0
	S3	1187	1275	28532	736	18270	0	2.374	2.55	57.064	1.472	36.54	0
SSLR	S1	4691	1345	24860	414	18690	0	9.382	2.69	49.72	0.828	37.38	0
	S2	4784	1438	24249	543	18986	0	9.568	2.876	48.498	1.086	37.972	0
	S3	4686	1537	24117	600	19060	0	9.372	3.074	48.234	1.2	38.12	0
xGen	S1	8308	964	17252	682	22794	0	16.616	1.928	34.504	1.364	45.588	0
	S2	8146	1006	17484	688	22676	0	16.292	2.012	34.968	1.376	45.352	0
	S3	7906	987	17986	617	22504	0	15.812	1.974	35.972	1.234	45.008	0

Table S8

Biome	Library	Sample	Total reads for virome	Virgaviridae	Herelleviridae	Herpesviridae	Intestiviridae	Microviridae	Mimiviridae	Other	Suoliviridae	Unclassified	Unknown	overall alignment rate
Fecal samples	MDA_0.5h	S1	3383390	3.99	0.02	0.10	0.17	2.45	0.16	0.55	4.56	77.17	10.82	46.01%
		S2	3681724	3.96	0.03	0.08	0.16	2.82	0.19	0.59	4.69	76.65	10.84	45.27%
		S3	5045494	4.02	0.03	0.09	0.14	2.64	0.20	0.59	4.66	77.16	10.47	45.41%
	MDA_1.5h	S1	4841122	3.93	0.01	0.11	0.09	1.95	0.30	0.52	3.77	78.56	10.76	47.10%
		S2	4681524	4.11	0.01	0.12	0.10	2.05	0.27	0.49	3.81	78.73	10.31	50.16%
		S3	2859504	4.42	0.01	0.10	0.11	1.69	0.27	0.49	4.05	79.06	9.81	51.47%
	Nextera	S1	2931482	0.92	0.00	0.10	0.02	0.13	0.31	1.19	0.71	66.70	29.93	18.58%
		S2	2238562	0.95	0.00	0.10	0.01	0.15	0.31	1.17	0.72	65.53	31.06	18.53%
		S3	3071094	0.93	0.00	0.10	0.01	0.13	0.32	1.17	0.76	66.40	30.18	18.72%
	SSLR	S1	5178132	2.76	0.03	0.10	0.01	0.14	0.61	0.89	0.81	73.51	21.14	13.45%
		S2	6459956	3.35	0.04	0.08	0.03	0.22	0.54	0.90	1.26	72.89	20.69	14.76%
		S3	5352486	3.44	0.03	0.08	0.02	0.22	0.56	0.82	1.36	72.89	20.58	15.31%
	xGen	S1	6881604	7.18	0.02	0.07	0.04	0.48	0.45	0.84	2.02	69.32	19.58	13.08%
		S2	6849666	6.96	0.03	0.07	0.04	0.46	0.47	0.91	2.03	69.22	19.79	13.07%
		S3	6772802	6.82	0.02	0.09	0.03	0.39	0.52	0.88	1.86	69.20	20.20	12.20%

Table S9

					Blast output							database_	Metadata																
Query	Target	Percent identit	Alignment	Number o	Number o	Start posit	End positi	Start posit	End positi	E-value	Bit score c	database	GPD_id	Source	GPD_VC	Size	Predicted_	Host_rang	Host_rang	Metageno	Continents	Countries_	checkV_MI	checkV_co	checkV_vir	checkV_ho	checkV_pr	checkV_te	Novel
NC_019521.1	ERR101823	89.1	55	6	0	1282	1336	25943	25997	1.50E-06	69.4	GPD	uvig_217495	Metageno	14888	54731	0	0	0	SRR413602	NA,Asia,Eu	NA,China,S	Genome-fr	20.8	1-54731	0	No	No	No
NC_019521.1	ERR414345	85.1	74	11	0	1352	1425	6621	6694	8.70E-09	76.8	GPD	uvig_67118	Metageno	3594	61188	0	0	0	ERR414345	Europe	Denmark	Genome-fr	23.59	1-61188	0	No	No	Yes
NC_019521.1	ERR321602	91	67	6	0	1601	1667	33495	33561	3.10E-13	91.6	GPD	uvig_47967	Metageno	1577	65330	0	0	0	ERR321602	Europe,Asi	Denmark,C	Genome-fr	37.25	1-65330	0	No	No	Yes
NC_019521.1	ERR912194	74	169	44	0	139858	140026	34944	35112	1.50E-06	69.4	GPD	uvig_179207	Metageno	29414	92726	0	0	0	ERR912194	Europe	United Kin	High-quali	100	44726-927	1-44725	Yes	No	Yes
NC_019521.1	SRR505679	84.1	88	14	0	173244	173331	8117	8030	1.50E-11	86.1	GPD	uvig_460396	Metageno	34183	17754	0	0	0	SRR505673	North Ame	United Sta	Genome-fr	5.68	1-15216	15217-177	Yes	No	Yes
NC_019521.1	SRR555811	80.4	97	19	0	173497	173593	115561	115465	3.10E-08	75	GPD	uvig_568562	Metageno	27148	139085	0	0	0	SRR555800	Europe,Eu	Spain,Spai	High-quali	90.03	1-139085	0	No	No	Yes
NC_019521.1	ERR119093	78.4	116	25	0	217104	217219	25216	25331	8.70E-09	76.8	GPD	uvig_255336	Metageno	854	100967	0	GCA_0034	Bacteroidc	ERR119093	Asia,Asia	China,Chir	High-quali	95.37	1-100967	0	No	No	No

Query	Target	Percent identit	Alignment	Number o	Number o	Start posit	End positi	Start posit	End positi	E-value	Bit score c	database	Contig	Study	Nucleic_Ac	Eukaryotic	Length	Circular_St	Prophage	Prophage_	VirSorter_	VirFinder_	VirFinder_	Total_Con	NR_Protei	CAT_data	No_BUSCO	BUSCO_Ra	No_VPF_Hits
NC_019521.1	Shkoporov	83.8	74	12	0	1352	1425	7800	7727	0.000000045	71.3	GVD	Shkoporov_2019_SRR9161	Shkoporov	dsDNA	Bacterioph	7873	0	No	0	2	0.222	0.205918	10	8	Bacteria: 1	0	0	1
NC_019521.1	Han_2018_	93	57	4	0	1605	1661	9	65	5.8E-12	84.2	GVD	Han_2018_ERR1398079_N(	Han_2018	dsDNA	Bacterioph	57466	circular	Yes	circular_ge	6	0.943	0.00825	166	89	Bacteria: 0	1	0.006024	189
NC_019521.1	Ma_2019_	77.6	107	24	0	139439	139545	1081	1187	0.0000021	65.8	GVD	Ma_2019_SRR341609_NOD	Ma_2019	dsDNA	Bacterioph	5078	0	No	0	2	0.109	0.321	7	7	Bacteria: 1	0	0	0
NC_019521.1	Han_2018_	77.3	119	27	0	139904	140022	546	664	0.000000045	71.3	GVD	Han_2018_ERR1398160_N(	Han_2018	dsDNA	Bacterioph	6898	0	No	0	2	0.0752	0.385278	12	11	Bacteria: 0	0	0	2
NC_019521.1	Zuo_2017_	75.4	240	59	0	173169	173408	4778	5017	5.7E-22	117.5	GVD	Zuo_2017_SRR5677784_NC	#N/A	#N/A	#N/A	#N/A	#N/A	#N/A	#N/A	#N/A	#N/A	#N/A	#N/A	#N/A	#N/A	#N/A	#N/A	#N/A
NC_019521.1	Ma_2019_	75.4	134	33	0	217086	217219	46295	46162	0.0000021	65.8	GVD	Ma_2019_SRR341682_NOD	Ma_2019	dsDNA	Bacterioph	29494	0	Yes	0-29494	6	0.97	0.00521	162	65	Bacteria: 0	1	0.006173	1

Query	Target	Percent identit	Alignment	Number o	Number o	Start posit	End positi	Start posit	End positi	E-value	Bit score c	database	contig_id	assembly_	assembly_	study_acc	sample_ac	run_access	continent	country_c	sex	age	health	disease		contig_id	votu_id	length	checkv_qu	prophage	temperate	virulent_s	completen	gc	stop_codo	baltimore	ictv_order	ictv_family	ictv_genus
NC_019521.1	MGV-GEN(	91.5	59	5	0	1280	1338	140983	141041	3.1E-10	82.4	MGV	MGV-GENOME-0377247	EBI	ERR589771	ERP006678	ERS537306	ERR589771	Asia	CHN	male	59	NULL	NULL		MGV-GEN(	OTU-49059	163708	High-quali	No	0.054956	0.945044	100	28.9534	TGA	dsDNA	Caudoviral	NULL	NULL
NC_019521.1	MGV-GEN(	84.8	79	12	0	1347	1425	15141	15063	1.1E-09	80.5	MGV	MGV-GENOME-0094053	CIBIO	BackhedF_	ERP005989	ERS473224	ERR525889	Europe	SWE	female	31	NULL	NULL		MGV-GEN(	OTU-28578	15193	High-quali	No	1	0	100	52.3267	NULL	dsDNA	Caudoviral	Siphovirida	NULL
NC_019521.1	MGV-GEN(	91	67	6	0	1601	1667	116436	116502	5.1E-13	91.6	MGV	MGV-GENOME-0375650	EBI	ERR321602	ERP003612	ERS328953	ERR321602	Europe	DNK	NULL	NULL	NULL	NULL		MGV-GEN(	OTU-0145(	148358	Complete	No	0.1625	0.8375	84.58	32.6615	NULL	dsDNA	Caudoviral	NULL	NULL
NC_019521.1	MGV-GEN(	73.2	429	115	0	173169	173597	42684	43112	1.8E-32	156.2	MGV	MGV-GENOME-0355466	CIBIO	BritoIL_20	SRP029441	SRS475705	SRR22506(	Oceania	FJI	NULL	NULL	NULL	NULL		MGV-GEN(	CIBIO	BritoIL_20	SRP029441	SRS475705	SRR22506(	Oceania	FJI	NULL	NULL	NULL	NULL		
NC_019521.1	MGV-GEN(	78.4	116	25	0	217104	217219	36692	36807	0.000000014	76.8	MGV	MGV-GENOME-0366655	CIBIO	HeQ_2017_	ERP017091	ERS134338	ERR162032	Asia	CHN	NULL	0.08	NULL	NULL		MGV-GEN(	CIBIO	HeQ_2017_	ERP017091	ERS134338	ERR162032	Asia	CHN	NULL	0.08	NULL	NULL		

Query	Target	Percent identit	Alignment	Number o	Number o	Start posit	End positi	Start posit	End positi	E-value	Bit score c	database																		
query	target	fident	alnlen	mismatch	gapopen	qstart	qend	tstart	tend	evalue	bits	UVIG	Taxon_oid	Scaffold_o	Coordinat	Ecosystem	vOTU	Length	Topology	geNomad	Confidenc	Estimated	Estimated	MIUViG qu	Gene cont	Taxonomi	Taxonomi	Host taxor	Host predi	Sequence origin (doi)
NC_019521.1	IMGVR_UV	100	10000	0	0	20001	30000	20001	30000	0.00E+00	17918	IMGVR4.1_	IMGVR_UViG_2595698595_	2.6E+09	2.6E+09	whole	;;;	vOTU_086	219372	Linear	0.9804	High-confi	100	0	Reference	302;295;7;	r__Duplod	RefSeq	;;;;;;		RefSeq r213 (10.1093/nar/gkv1189)
NC_019521.1	IMGVR_UV	100	10000	0	0	30001	40000	30001	40000	0.00E+00	17918	IMGVR4.1_	IMGVR_UViG_2595698595_	2.6E+09	2.6E+09	whole	;;;	vOTU_086	219372	Linear	0.9804	High-confi	100	0	Reference	302;295;7;	r__Duplod	RefSeq	;;;;;;		RefSeq r213 (10.1093/nar/gkv1189)
NC_019521.1	IMGVR_UV	100	10000	0	0	40001	50000	40001	50000	0.00E+00	17918	IMGVR4.1_	IMGVR_UViG_2595698595_	2.6E+09	2.6E+09	whole	;;;	vOTU_086	219372	Linear	0.9804	High-confi	100	0	Reference	302;295;7;	r__Duplod	RefSeq	;;;;;;		RefSeq r213 (10.1093/nar/gkv1189)
NC_019521.1	IMGVR_UV	100	10000	0	0	140001	150000	140001	150000	0.00E+00	17918	IMGVR4.1_	IMGVR_UViG_2595698595_	2.6E+09	2.6E+09	whole	;;;	vOTU_086	219372	Linear	0.9804	High-confi	100	0	Reference	302;295;7;	r__Duplod	RefSeq	;;;;;;		RefSeq r213 (10.1093/nar/gkv1189)
NC_019521.1	IMGVR_UV	100	10000	0	0	150001	160000	150001	160000	0.00E+00	17918	IMGVR4.1_	IMGVR_UViG_2595698595_	2.6E+09	2.6E+09	whole	;;;	vOTU_086	219372	Linear	0.9804	High-confi	100	0	Reference	302;295;7;	r__Duplod	RefSeq	;;;;;;		RefSeq r213 (10.1093/nar/gkv1189)
NC_019521.1	IMGVR_UV	100	10000	0	0	80001	90000	80001	90000	0.00E+00	17918	IMGVR4.1_	IMGVR_UViG_2595698595_	2.6E+09	2.6E+09	whole	;;;	vOTU_086	219372	Linear	0.9804	High-confi	100	0	Reference	302;295;7;	r__Duplod	RefSeq	;;;;;;		RefSeq r213 (10.1093/nar/gkv1189)
NC_019521.1	IMGVR_UV	73.1	626	168	0	179248	178623	42028	42653	4.60E-93	356	IMGVR4.1_	IMGVR_UViG_2772190697_	2.77E+09	2.77E+09	29560-756	Host-assoc	vOTU_085	46139	Provirus	0.9232	High-confi	100	0	High-quali	67;67;0;30	r__Duplod	geNomad	d__Bacteri	Isolate tax	geNomad v1.1.0 (10.5281/zenodo.7015982)
NC_019521.1	IMGVR_UV	73.1	626	168	0	179248	178623	42028	42653	4.60E-93	356	IMGVR4.1_	IMGVR_UViG_2772190700_	2.77E+09	2.77E+09	29873-760	Host-assoc	vOTU_085	46139	Provirus	0.9233	High-confi	100	0	High-quali	67;67;0;30	r__Duplod	geNomad	d__Bacteri	Isolate tax	geNomad v1.1.0 (10.5281/zenodo.7015982)
NC_019521.1	IMGVR_UV	73.1	626	168	0	179248	178623	43727	44352	4.60E-93	356	IMGVR4.1_	IMGVR_UViG_2772190702_	2.77E+09	2.77E+09	29785-776	;;;	vOTU_085	47838	Provirus	0.9334	High-confi	100	0	High-quali	70;70;0;31	r__Duplod	geNomad	d__Bacteri	Isolate tax	geNomad v1.1.0 (10.5281/zenodo.7015982)
NC_019521.1	IMGVR_UV	67.8	923	296	0	178619	179539	7059	7981	6.98E-77	302	IMGVR4.1_	IMGVR_UViG_3300014815_	3.3E+09	Ga0119815	4295-4890	Host-assoc	vOTU_028	44611	Provirus	0.9523	High-confi	100	0	High-quali	74;74;0;37	r__Duplod	geNomad	d__Bacteri	CRISPR spa	geNomad v1.1.0 (10.5281/zenodo.7015982)
NC_019521.1	IMGVR_UV	71.8	566	159	0	173168	173733	1058	1623	2.07E-70	281	IMGVR4.1_	IMGVR_UViG_3300029904_	3.3E+09	Ga0247319	whole	Host-assoc	vOTU_051	49606	Linear	0.9674	High-confi	34.44	0	Genome fr	85;85;0;20	r__Duplod	geNomad	;;;;;;		geNomad v1.1.0 (10.5281/zenodo.7015982)
NC_019521.1	IMGVR_UV	72	566	158	0	173168	173733	42683	43248	3.86E-70	280	IMGVR4.1_	IMGVR_UViG_3300045988_	3.3E+09	Ga0495776	whole	Host-assoc	vOTU_079	84755	Linear	0.9726	High-confi	77.62	0	Genome fr	116;114;2;	r__Duplod	geNomad	;;;;;;		geNomad v1.1.0 (10.5281/zenodo.7015982)

Table S10

Phage	DSM	Host	Strain ID	DSM_host	Temp. (°C)	Medium	Modification site (possible)	Source / reference
**C2**	In house	Lactococcus lactis	MG1363	DSM 4366	30	M17+0.5% glucose/5 mM $CaCl_2$	**Possible (unknown)**	Lab stock
**T4**	16352	Escherichia coli	Luria	DSM 613	37	NB (7.0)/LB (shaking)	**5-HMC/glc-HMC**	Lab stock
**T4-c**	--	Escherichia coli (Migula 1895) Castellani and Chalmers 1919	L17	DSM 6254	37	NB (7.0)/LB (shaking)	**5-HMC/glc-HMC**	Lab stock
**Phi29**	5546	Bacillus subtilis	Ehrenberg 1835	DSM 5547	37	TSB	Unknown	Lab stock
**P1**	5757	Escherichia coli	MG1655	DSM 18039	37	LB + 10 mM $CaCl_2$ & $MgSO_4$ (shaking)	Possible (unknown)	Lab stock
**T7**	4623	Escherichia coli	MG1655	DSM 18039	37	LB (shaking)	Unknown	Lab stock
**P35**	In house	Listeria monocytogenes	DP-L861	Mack	30	½ BHI	Unknown	Kilcher et al,. 2018
**Lambda**	4499	Escherichia coli	Migula 1895	DSM 4230	37	LB	Unknown	Thermo Scientific (Catalog #:SD0011)
**Phi X174**	4497	Escherichia coli	PC 0886	DSM 13127/ATCC 13706	37	BHI+10 mM $CaCl_2$ & $MgSO_4$ (shaking)	Unknown	NEB (Catalog #:N3023S)
**M13mp18**	5919	Escherichia coli	ER2738	DSM 3413	37	LB	Unknown	NEB (Catalog #:N4040S)
**MS2**	13767	Escherichia coli	W1485	DSM 5695	37	NZCYM (pH7.5) + 2mg/L streptomycin	Unknown	Lab stock
**Phi6**	21518	Pseudomonas sp.	HER 1102	DSM 21482	25	TSB (Ph7.3) + 0.25% glucose (shaking)	Unknown	Lab stock

Table S11

Mock	Library_method/ Treatment	Alignment rate (%)	
		With T4	No T4
A	MDA_0.5h	86.2	86.53
	MDA_1.5h	86.95	87.31
	Nextera	90.55	91.04
	SSLR	86.39	86.39
	xGen	90.23	89.22
B	MDA_0.5h	88.53	89.18
	MDA_1.5h	90.15	89.76
	Nextera	90.87	91.23
	SSLR	84.3	86.64
	xGen	92.07	91.34
C	MDA_0.5h	89.4	90.52
	MDA_1.5h	90.83	91.07
	Nextera	90.67	90.78
	SSLR	85.37	83.68
	xGen	95.03	95.17
D	DMSO		6.15
	Heat		66.86
	No DMSO		4.37
	No Heat		46.22
E	DMSO		6.02
	Heat		61.94
	No DMSO		7.18
	No Heat		76.3
F	DMSO		15.55
	Heat		77.97
	No DMSO		7.09
	No Heat		86.93
G (T4)	MDA_0.5h	88.62	
	MDA_1.5h	91.09	
	Nextera	94.48	
	SSLR	92.31	
	xGen	95.79	
H (T4-c)	MDA_0.5h	89.77	
	MDA_1.5h	89.55	
	Nextera	95.14	
	SSLR	93.67	
	xGen	95.25	
