# Supplementary material for: A single-stranded based library preparation method for virome characterization
Source: Microbiome. 2024 Oct 24;12:219. doi: 10.1186/s40168-024-01935-5 (PMC11515303; doi:10.1186/s40168-024-01935-5)
Supplement: Supplementary file 6 — Additional file 5. Supplementary Figures S1-S10. Fig. S1 | Virome sequencing library preparation strategy with different types of nucleic acids (dsDNA, ssDNA, dsRNA and ssRNA). (A) Workflow for DNA phage genomes (dsDNA and ssDNA) library preparation with different library methods to varying ratios of genomes (Mock A, Mock B and Mock C). (B) Workflow for DNA and RNA phage genomes library preparation with the single-stranded library at different ratios of genomes (Mock D, Mock E and Mock F). (C) Workflow for comparison of the modification existence for (Mock G with T4, Mock H with T4-c), where T4 is a heavily modified genome and T4-c is a lower modified genome. (D) Workflow for comparison of the fecal sample (Fecal virome: FV) with different library preparations. (E) Percentage of phage genomes of DNAs library with different ratios of ssDNA and dsDNA phage genomes, three artificial virome communities (Mock A, Mock B and Mock C) containing different proportions of the ssDNA phage phiX174 and M13mp18 mixed with dsDNA phage. These phage genome abundance values were calculated based on the quantity of dsDNA and ssDNA phages measured Qubit dsDNA (or ssDNA) HS Assay kit. Mock A: ratio of ~90:10 for dsDNA and ssDNA; Mock B: ratio of ~50:50 for dsDNA and ssDNA; Mock C: ratio of ~50:50 for dsDNA and ssDNA. (F) Percentage of phage genomes of DNA/RNAs library with different ratios of DNA and RNA phage genomes, three artificial viromes containing different proportions of the dsRNA phage Phi6 and ssRNA MS2 mixed with DNA phage. These phage genome abundance values were calculated based on the quantity of DNA and RNA phages measured with Nanodrop1000. Mock D: ratio of 90:10 for DNA and RNA; Mock E: ratio of 50:50 for DNA and RNA; Mock F: ratio of 10:90 for DNA and RNA. (G) Percentage of phage genomes of DNAs library with 2 different T4 genomes, where MG: Mock G containing high modification T4 genome (T4) with equal ratio of all genomes; MH: Mock H containing lower modification T4 ge [file 40168_2024_1935_MOESM5_ESM.pdf]

Supplementary figures for the SSLR paper entitled “ A single strand-based library preparation method for virome characterization”

By

Xichuan Zhai, Alex Gobbi, Witold Kot, Lukasz Krych, Dennis Sandris Nielsen, Ling Deng

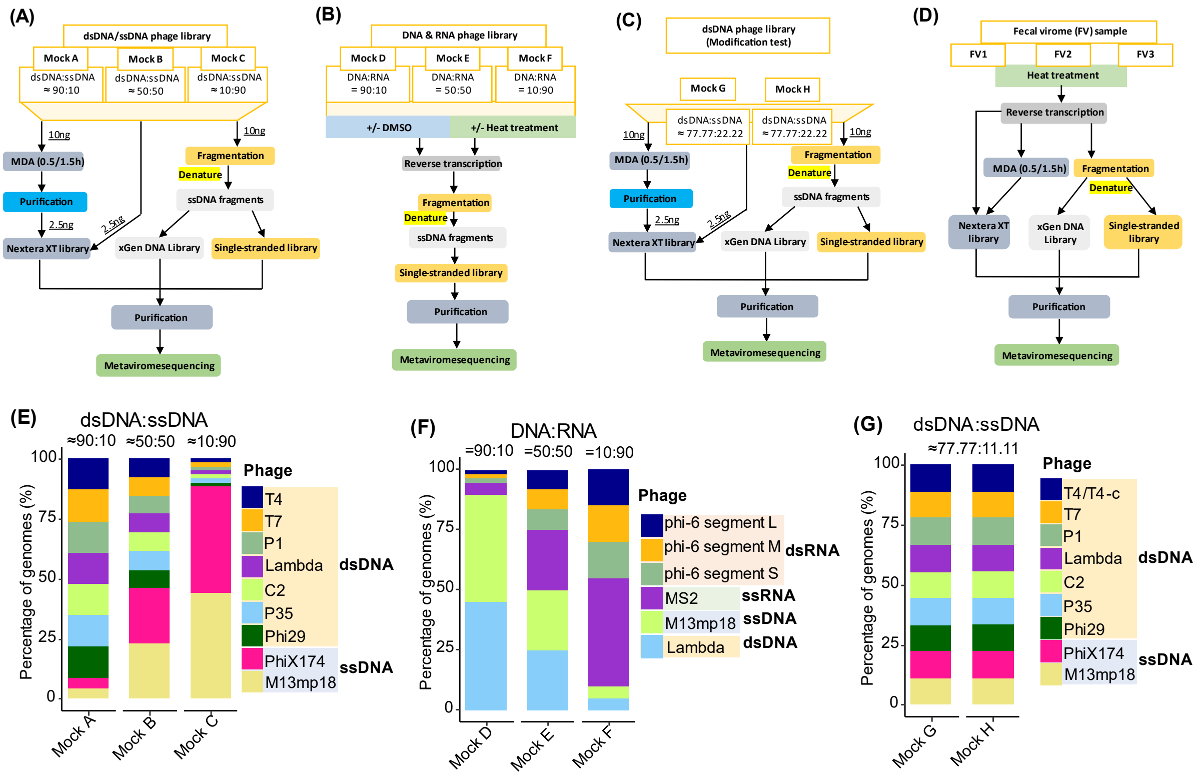

Fig. S1 | Virome sequencing library preparation strategy with different types of nucleic acids (dsDNA, ssDNA, dsRNA and ssRNA). (A) Workflow for DNA phage genomes (dsDNA and ssDNA) library preparation with different library methods to varying ratios of genomes (Mock A, Mock B and Mock C). (B) Workflow for DNA and RNA phage genomes library preparation with the single-stranded library at different ratios of genomes (Mock D, Mock E and Mock F). (C) Workflow for comparison of the modification existence for (Mock G with T4, Mock H with T4-c), where T4 is a heavily modified genome and T4-c is a lower modified genome. (D) Workflow for comparison of the fecal sample (Fecal virome: FV) with different library preparations. (E) Percentage of phage genomes of DNAs library with different ratios of ssDNA and dsDNA phage genomes, three artificial virome communities (Mock A, Mock B and Mock C) containing different proportions of the ssDNA phage PhiX174 and M13mp18 mixed with dsDNA phage. These phage genome abundance values were calculated based on the quantity of dsDNA and ssDNA phages measured Qubit dsDNA (or ssDNA) HS Assay kit. Mock A: ratio of ~90:10 for dsDNA and ssDNA; Mock B: ratio of ~50:50 for dsDNA and ssDNA; Mock C: ratio of ~50:50 for dsDNA and ssDNA. (F) Percentage of phage genomes of DNA/RNAs library with different ratios of DNA and RNA phage genomes, three artificial viromes containing different proportions of the dsRNA phage Phi6 and ssRNA MS2 mixed with DNA phage. These phage genome abundance values were calculated based on the quantity of DNA and RNA phages measured with Nanodrop1000. Mock D: ratio of 90:10 for DNA and RNA; Mock E: ratio of 50:50 for DNA and RNA; Mock F: ratio of 10:90 for DNA and RNA. (G) Percentage of phage genomes of DNAs library with 2 different T4 genomes, where MG: Mock G containing high modification T4 genome (T4) with equal ratio of all genomes; MH: Mock H containing lower modification T4 genome (T4-c) with equal ratio of all genomes.

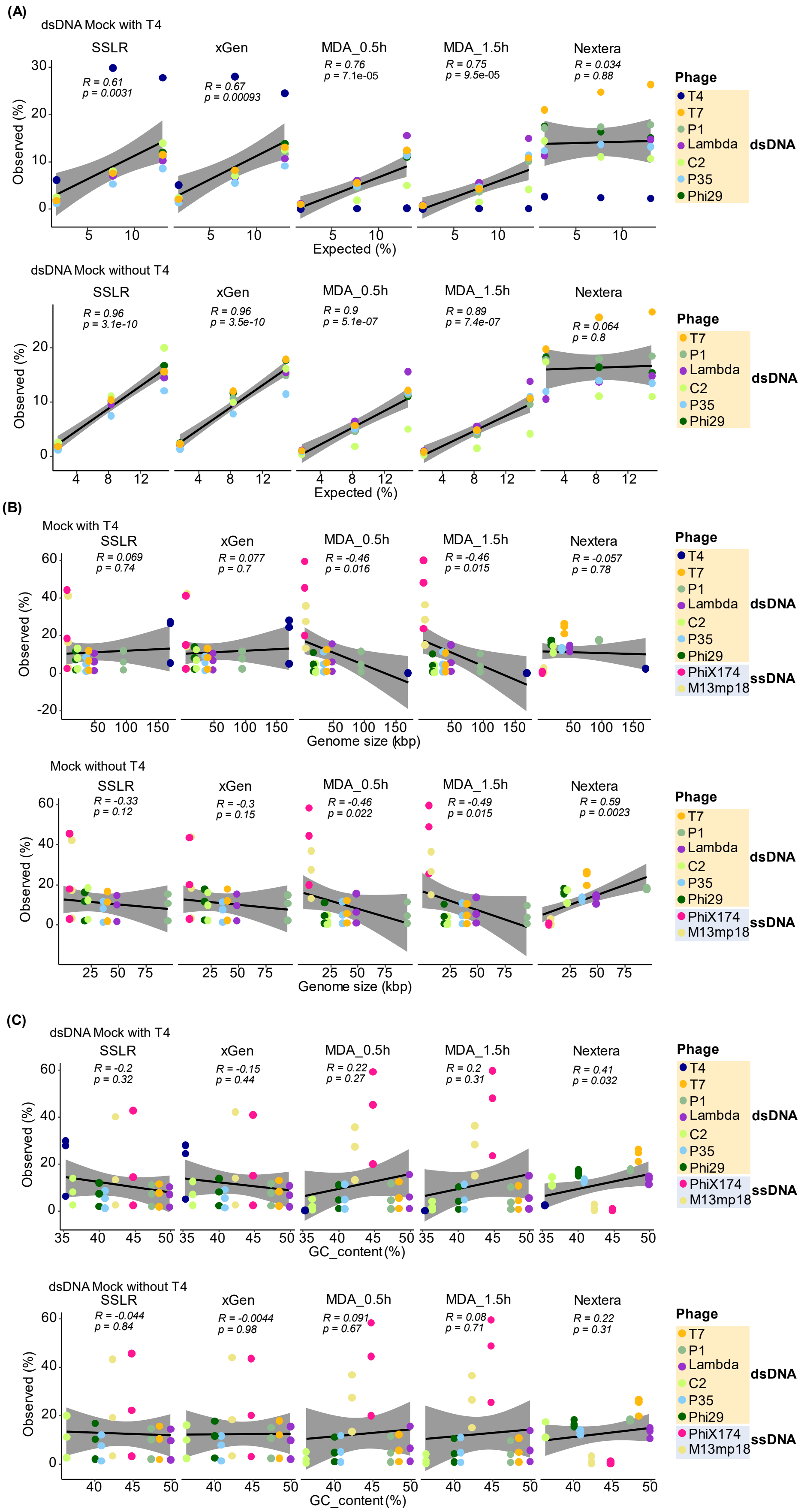
(A)
dsDNA Mock with T4
SSLR
R = 0.61
p = 0.0031
xGen
R = 0.67
p = 0.00093
MDA_0.5h
R = 0.76
p = 7.1e-05
MDA_1.5h
R = 0.75
p = 9.5e-05
Nextera
R = 0.034
p = 0.88
Observed (%)
Expected (%)
dsDNA Mock without T4
SSLR
R = 0.96
p = 3.1e-10
xGen
R = 0.96
p = 3.5e-10
MDA_0.5h
R = 0.9
p = 5.1e-07
MDA_1.5h
R = 0.89
p = 7.4e-07
Nextera
R = 0.064
p = 0.8
Observed (%)
Expected (%)
(B)
Mock with T4
SSLR
R = 0.069
p = 0.74
xGen
R = 0.077
p = 0.7
MDA_0.5h
R = -0.46
p = 0.016
MDA_1.5h
R = -0.46
p = 0.015
Nextera
R = -0.057
p = 0.78
Observed (%)
Genome size (kbp)
Mock without T4
SSLR
R = -0.33
p = 0.12
xGen
R = -0.3
p = 0.15
MDA_0.5h
R = -0.46
p = 0.022
MDA_1.5h
R = -0.49
p = 0.015
Nextera
R = 0.59
p = 0.0023
Observed (%)
Genome size (kbp)
(C)
dsDNA Mock with T4
SSLR
R = -0.2
p = 0.32
xGen
R = -0.15
p = 0.44
MDA_0.5h
R = 0.22
p = 0.27
MDA_1.5h
R = 0.2
p = 0.31
Nextera
R = 0.41
p = 0.032
Observed (%)
GC_content (%)
dsDNA Mock without T4
SSLR
R = -0.044
p = 0.84
xGen
R = -0.0044
p = 0.98
MDA_0.5h
R = 0.091
p = 0.67
MDA_1.5h
R = 0.08
p = 0.71
Nextera
R = 0.22
p = 0.31
Observed (%)
GC_content (%)
Phage
T4
T7
P1
Lambda
C2
P35
Phi29
dsDNA
PhiX174
M13mp18
ssDNA

Fig. S2 | Pearson correlation coefficient (r) and two-tailed p-value between the expected and obtained read distributions (in percentage) in different DNAs phage mock communities with or without phage T4 genome. (A) Pearson correlation only dsDNA phage genome with (Top panel) or without T4 (bottom panel) shows that the presence of T4 distorted the percentage of dsDNA phage genomes. The phage genome size (B) and GC_content (C) have minimal effect on the phage genome distributions for the library prepared with SSLR and xGen.

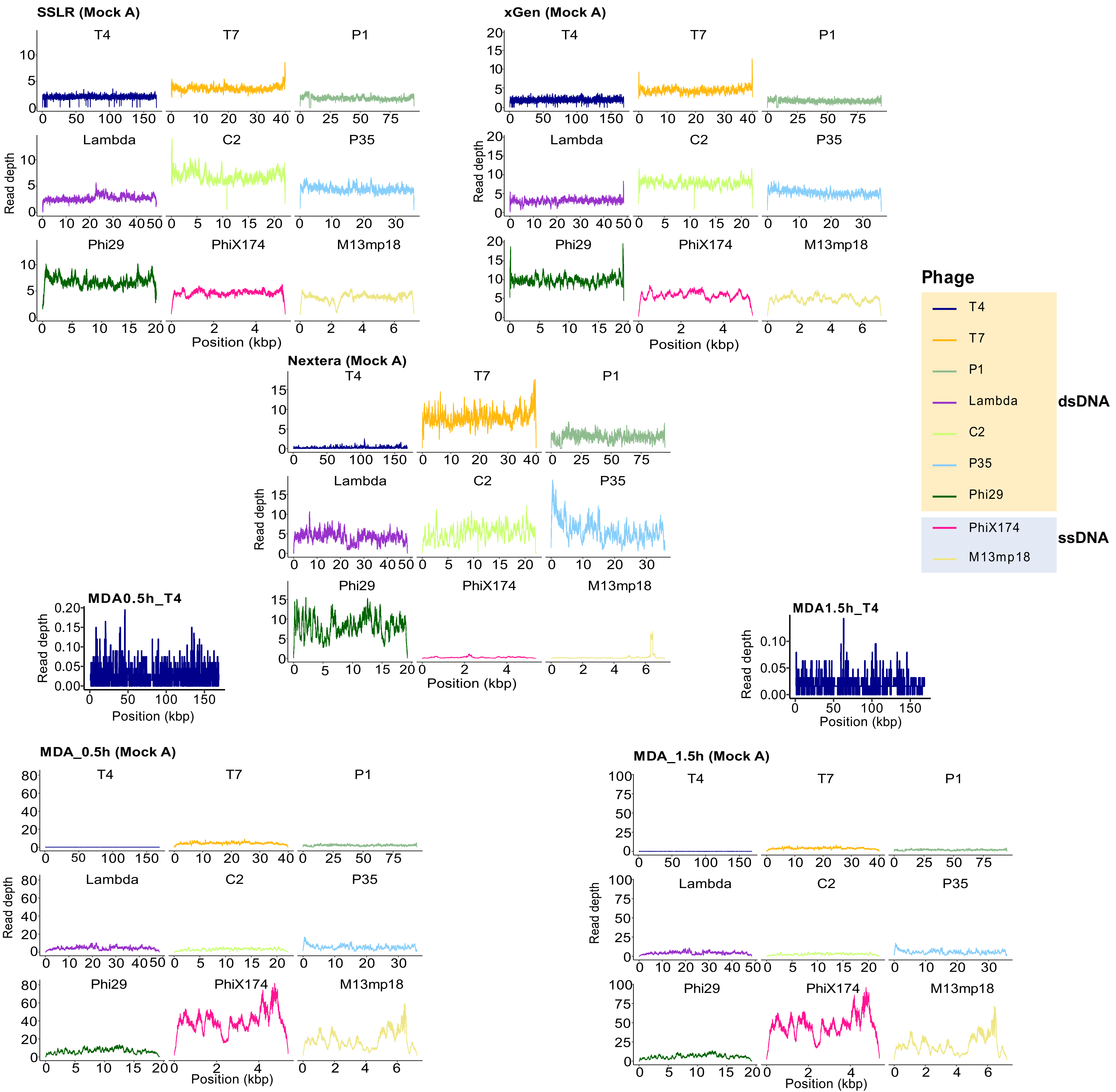

SSLR (Mock A)
xGen (Mock A)
Nextera (Mock A)
MDA0.5h_T4
MDA1.5h_T4
MDA_0.5h (Mock A)
MDA_1.5h (Mock A)
T4
T7
P1
Lambda
C2
P35
Phi29
PhiX174
M13mp18
Read depth
Position (kbp)
Phage
dsDNA
ssDNA

Fig. S3 | Different library methods induce different sequence coverages of the DNA phage genomes. Mock A was chosen for coverage visualization as it contains a high ratio of DNA genomes compared to Mock B and C. Read depth at each position in the phage genome was normalized by count/total reads*10000 for easier comparison among different library preparation methods. T4 genome amplified by MDA for 0.5 and 1.5 h resulted in low coverages.

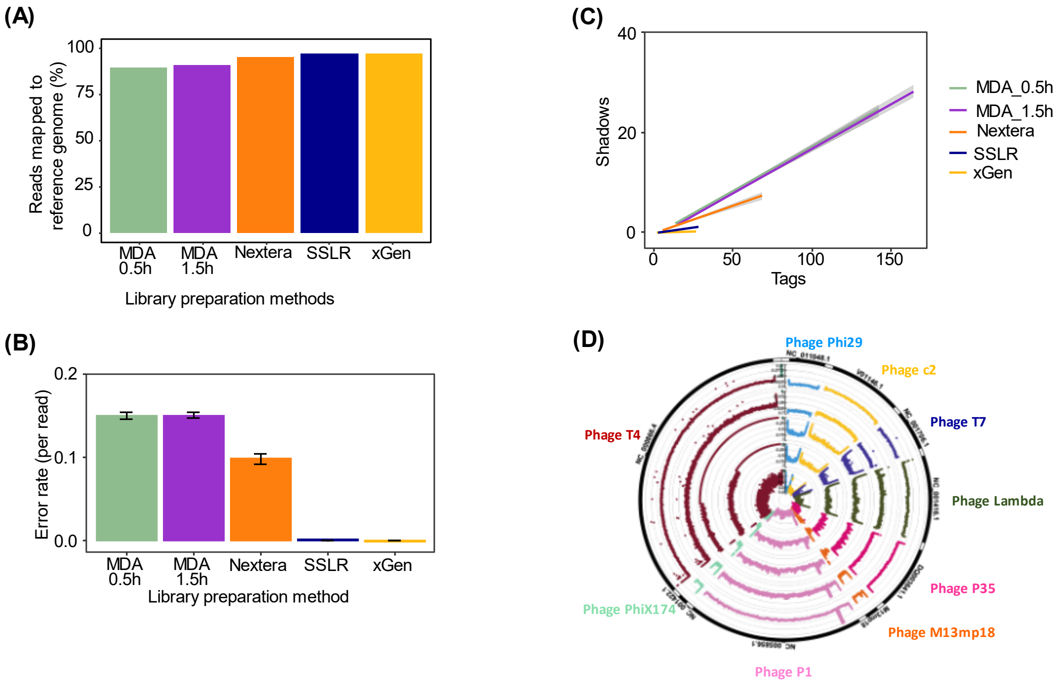

Fig. S4 | (A) The overall percentages of sequencing reads mapping to the 9 DNA reference genomes. The R package ShadowRegression estimates reference-free error rates (B) based on a transform of the slope of read counts and their 'shadows' (C). (D) Depth biases in the sequencing of DNA phage genomes prepared with different library methodsThe circle plot shows from the inside: Nextera (Ring 1); MDA_0.5h (Ring 2); MDA_1.5h (Ring 3); xGen (Ring 4) and SSLR (Ring 5). The circles are numbered from the inside. The sequencing depth was shown in the log10 scale. All the reads were normalized and then visualized using the script from https://github.com/padbr/gcbias.

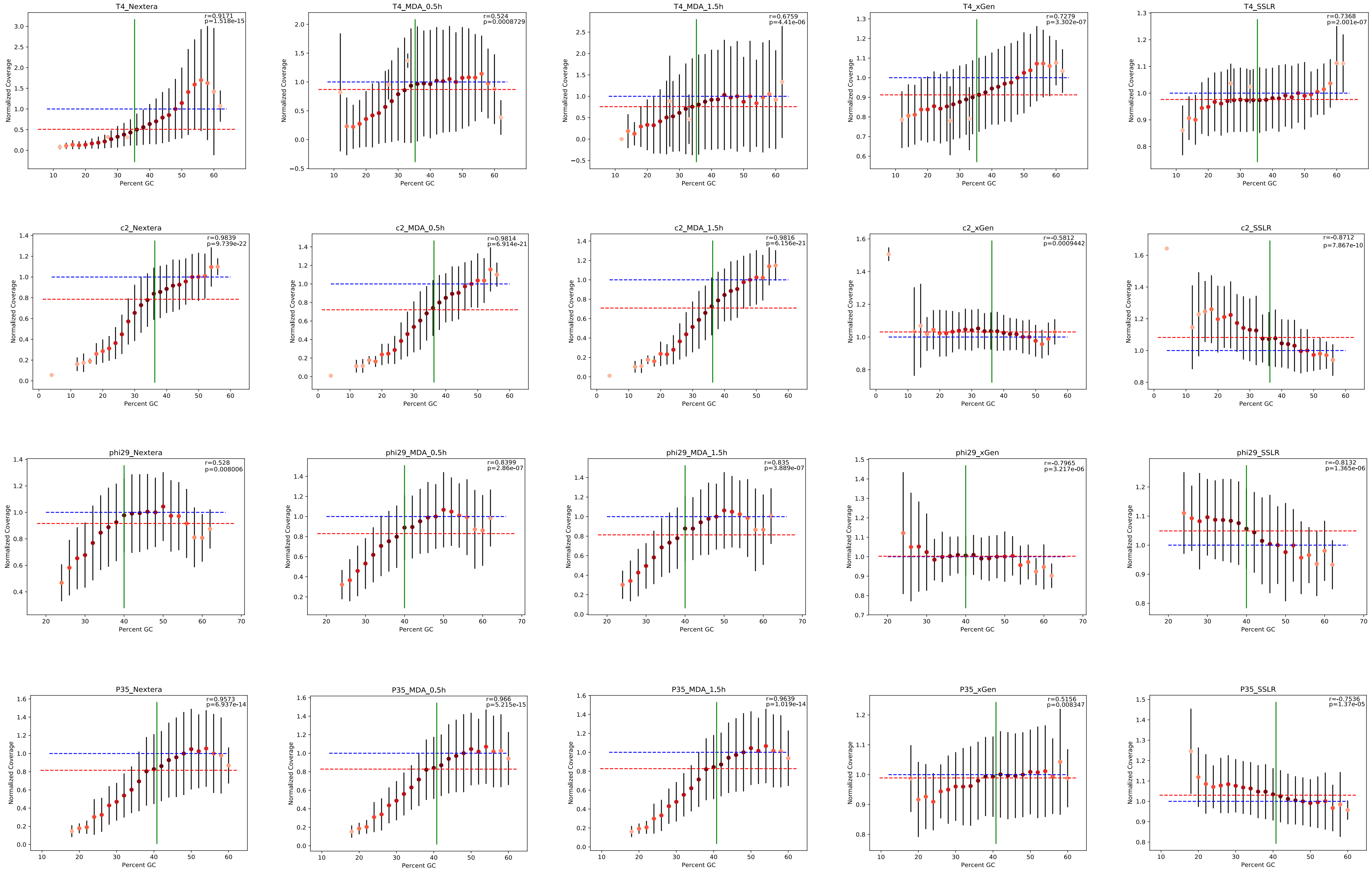

T4_Nextera
r=0.9171
p=1.518e-15
T4_MDA_0.5h
r=0.524
p=0.0008729
T4_MDA_1.5h
r=0.6759
p=4.41e-06
T4_xGen
r=0.7279
p=3.302e-07
T4_SSLR
r=0.7368
p=2.001e-07
c2_Nextera
r=0.9839
p=9.739e-22
c2_MDA_0.5h
r=0.9814
p=6.914e-21
c2_MDA_1.5h
r=0.9816
p=6.156e-21
c2_xGen
r=-0.5812
p=0.0009442
c2_SSLR
r=-0.8712
p=7.867e-10
phi29_Nextera
r=0.528
p=0.008006
phi29_MDA_0.5h
r=0.8399
p=2.86e-07
phi29_MDA_1.5h
r=0.835
p=3.889e-07
phi29_xGen
r=-0.7965
p=3.217e-06
phi29_SSLR
r=-0.8132
p=1.365e-06
P35_Nextera
r=0.9573
p=6.937e-14
P35_MDA_0.5h
r=0.966
p=5.215e-15
P35_MDA_1.5h
r=0.9639
p=1.019e-14
P35_xGen
r=0.5156
p=0.008347
P35_SSLR
r=-0.7536
p=1.37e-05
Normalized Coverage
Percent GC

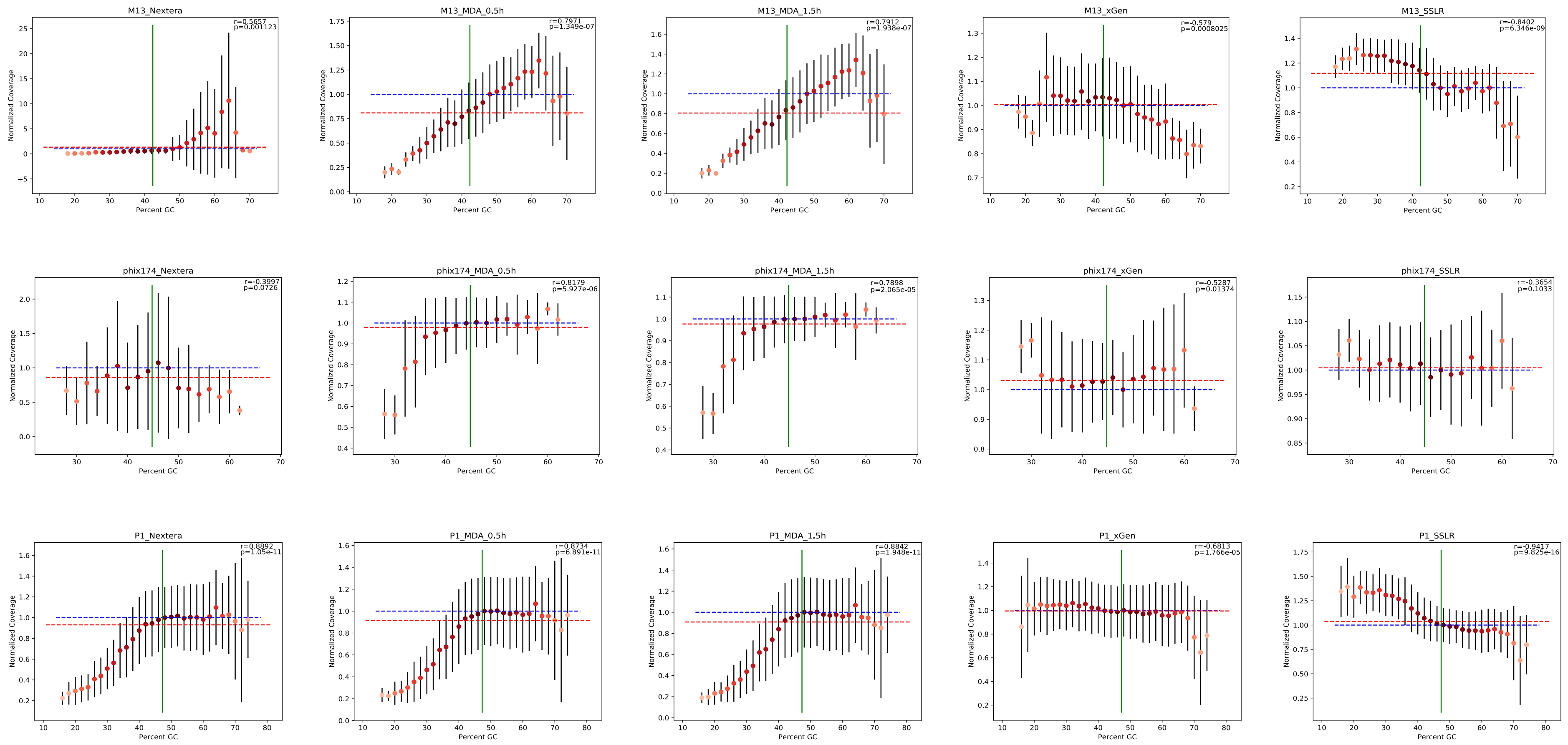
M13_Nextera
r=0.5657
p=0.001123
M13_MDA_0.5h
r=0.7971
p=1.349e-07
M13_MDA_1.5h
r=0.7912
p=1.938e-07
M13_xGen
r=-0.579
p=0.0008025
M13_SSLR
r=-0.8402
p=6.346e-09
phix174_Nextera
r=-0.3997
p=0.0726
phix174_MDA_0.5h
r=0.8179
p=5.927e-06
phix174_MDA_1.5h
r=0.7898
p=2.065e-05
phix174_xGen
r=-0.5287
p=0.01374
phix174_SSLR
r=-0.3654
p=0.1033
P1_Nextera
r=0.8892
p=1.05e-11
P1_MDA_0.5h
r=0.8734
p=6.891e-11
P1_MDA_1.5h
r=0.8842
p=1.948e-11
P1_xGen
r=-0.6813
p=1.766e-05
P1_SSLR
r=-0.9417
p=9.825e-16
Normalized Coverage
Percent GC

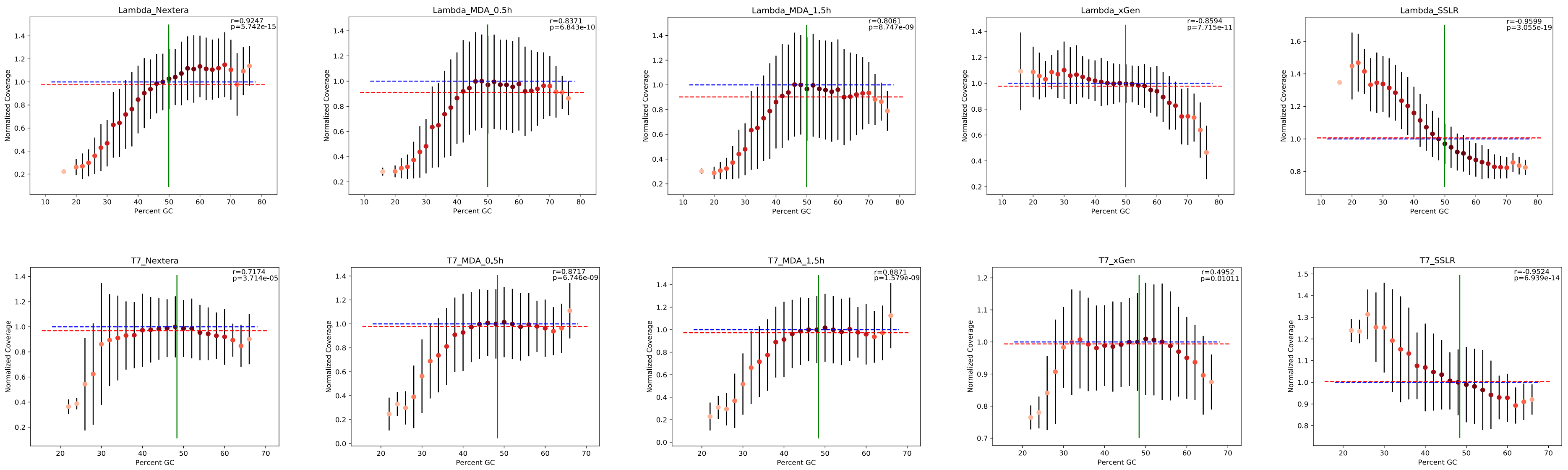

Fig. S5 | Coverage biases in datasets for the different DNA phage genomes with different GC contents. Dot plots show local GC content and normalized relative coverages in 50-nt windows of NextSeq/iSeq and data from a variety of phages with different average GC contents. Error bars indicate ±1 standard deviation of normalized coverage. The intensity of the blue in the dots is a log-transformed heat map of the number of 50-nt windows averaged into that data point. The data point with the most windows in each plot has maximum red. The vertical green line marks the average GC content of each assembly. The average normalized coverage value is indicated with a horizontal dashed red line. The plots were visualized with the Python script from: https://github.com/padbr/gcbias, with the modifications by adding the Pearson correlation coefficient (r), and two-tailed p-value (top right corner of each plot).

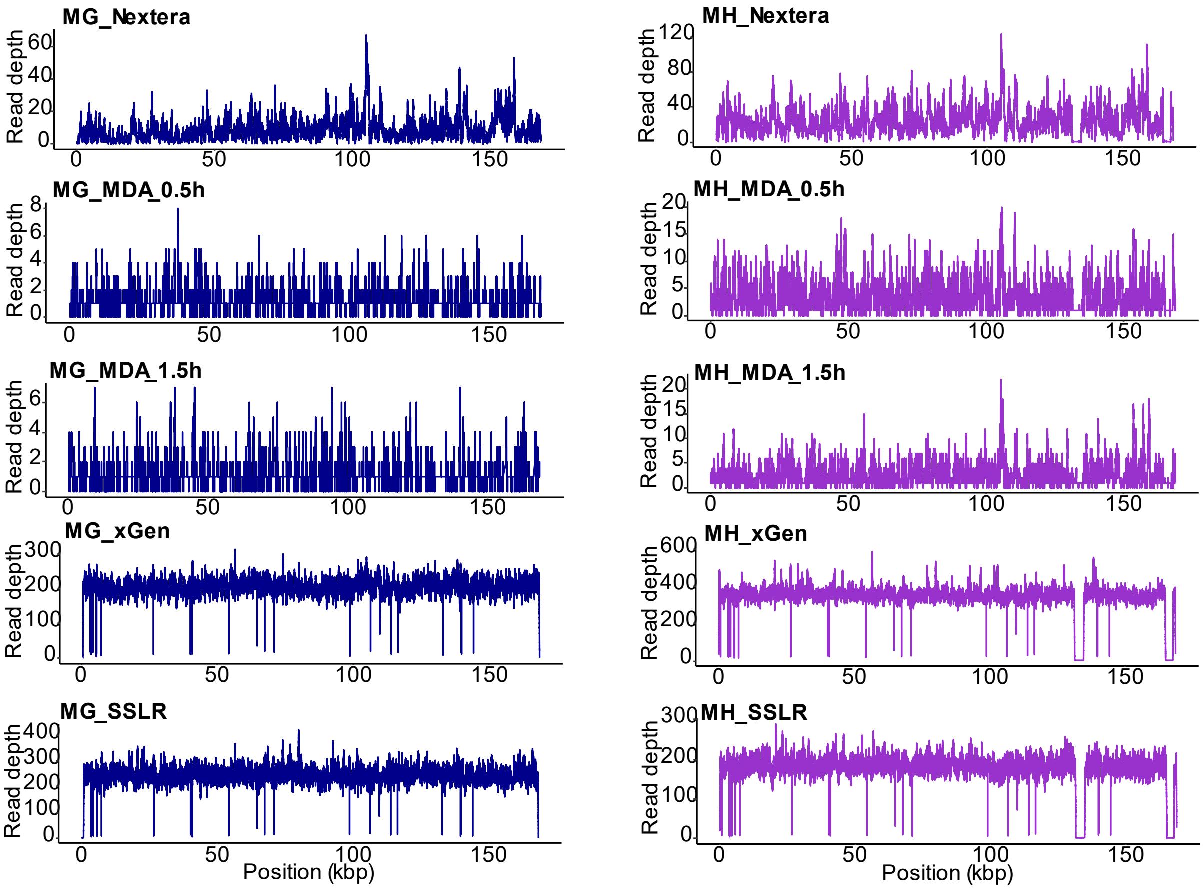

Fig. S6 | Different library methods induce different sequence coverages of the T4 (blue line)/T4-c (purple line) phage genomes. Read depth at each position in the phage genome was normalized by count/total reads*10000 for easier comparison among different library preparation methods.

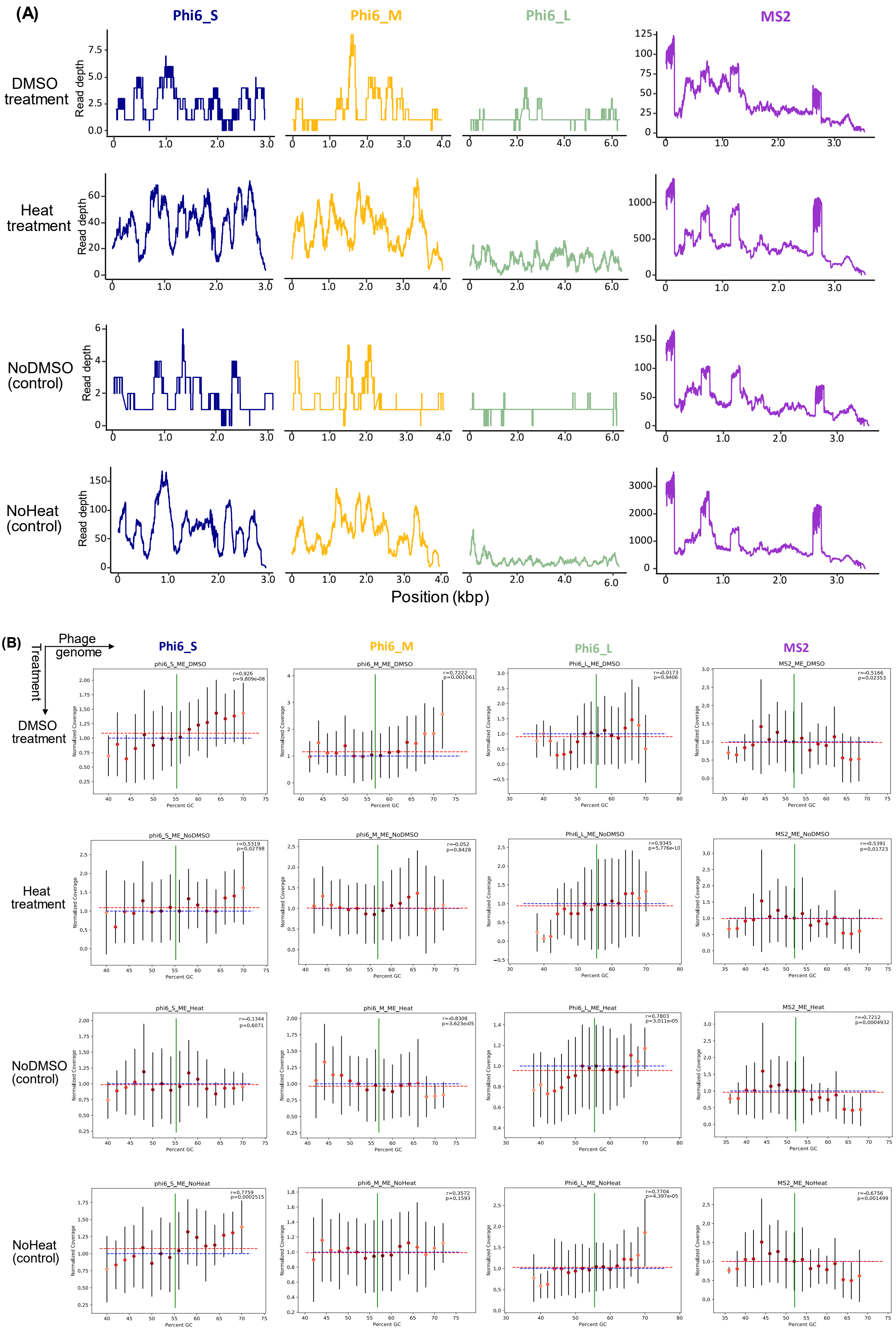
(A)
Phi6_S
Phi6_M
Phi6_L
MS2
DMSO treatment
Heat treatment
NoDMSO (control)
NoHeat (control)
Read depth
Position (kbp)
(B)
Phage genome
Treatment
phi6_S_ME_DMSO
r=0.926
p=9.809e-08
phi6_M_ME_DMSO
r=0.7222
p=0.001061
Phi6_L_ME_DMSO
r=-0.0173
p=0.9406
MS2_ME_DMSO
r=-0.5166
p=0.02353
phi6_S_ME_NoDMSO
r=0.5319
p=0.02798
phi6_M_ME_NoDMSO
r=-0.052
p=0.8428
Phi6_L_ME_NoDMSO
r=0.9345
p=5.776e-10
MS2_ME_NoDMSO
r=-0.5391
p=0.01723
phi6_S_ME_Heat
r=-0.1344
p=0.6071
phi6_M_ME_Heat
r=-0.8308
p=3.623e-05
Phi6_L_ME_Heat
r=0.7803
p=3.011e-05
MS2_ME_Heat
r=-0.7212
p=0.0004932
phi6_S_ME_NoHeat
r=0.7759
p=0.0002515
phi6_M_ME_NoHeat
r=0.3572
p=0.1593
Phi6_L_ME_NoHeat
r=0.7704
p=4.397e-05
MS2_ME_NoHeat
r=-0.6756
p=0.001499
Normalized Coverage
Percent GC

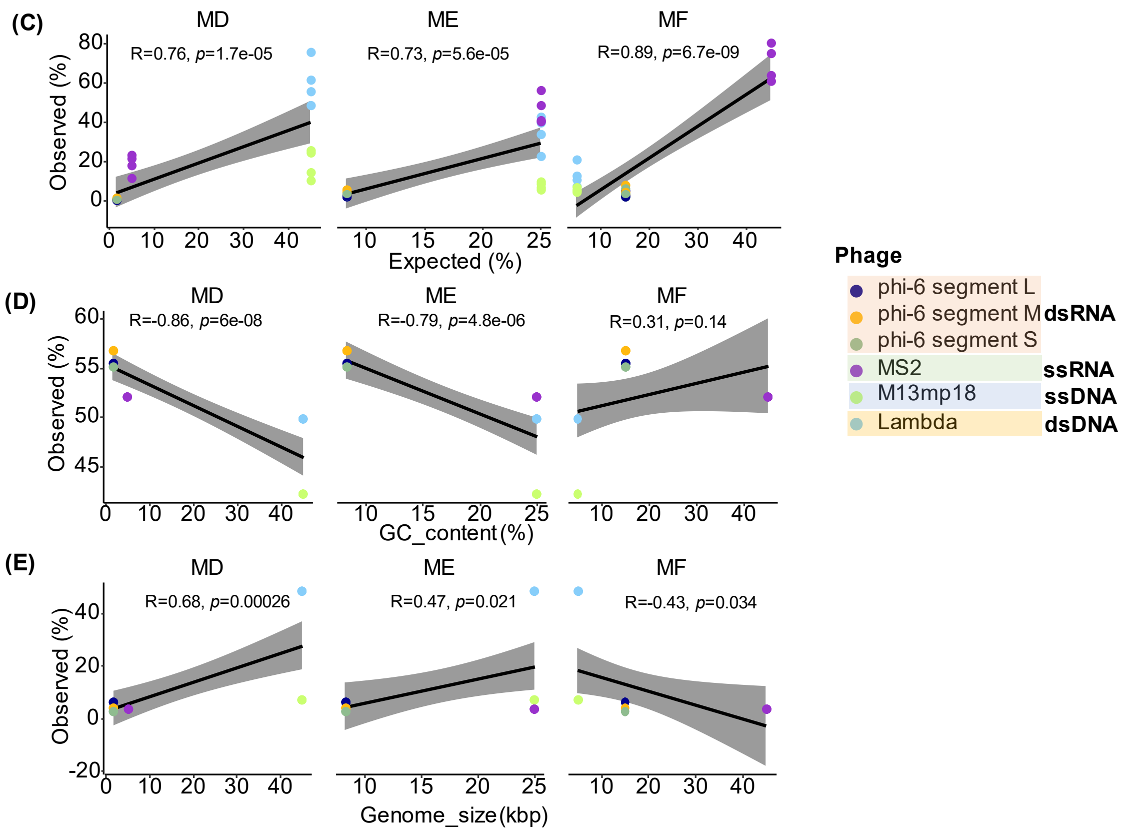

Fig. S7 | (A) Effect of DMSO and heat treatment on sequence coverage of the RNA phage genomes. Read depth at each position in the phage genomes under varying treatments (DMSO and Heat) and their controls (NoDMSO and NoHeat) are shown. (B) Coverage biases in datasets for the different types of RNA phage genomes with different GC contents. Dot plots show local GC content and normalized relative coverages in 50-nt windows and data from a variety of phages with different average GC contents. Error bars indicate ±1 standard deviation of normalized coverage. The intensity of the blue in the dots is a log-transformed heat map of the number of 50-nt windows averaged into that data point. The data point with the most windows in each plot has maximum red. The vertical green line marks the average GC content of each assembly. The average normalized coverage value is indicated with a horizontal dashed red line. The plots were visualized with the Python script from: https://github.com/padbr/gcbias, with the modifications by adding the Pearson correlation coefficient (r), and two-tailed p-value (top right corner of each plot). (C) Pearson correlation coefficient (r) and two-tailed p-value between the expected and obtained read distributions (in percentage) in different DNA/RNA phage mock communities. The phage genome size (D) and GC_content (E) have minimal effect on the phage genome distributions for the library prepared with SSLR.

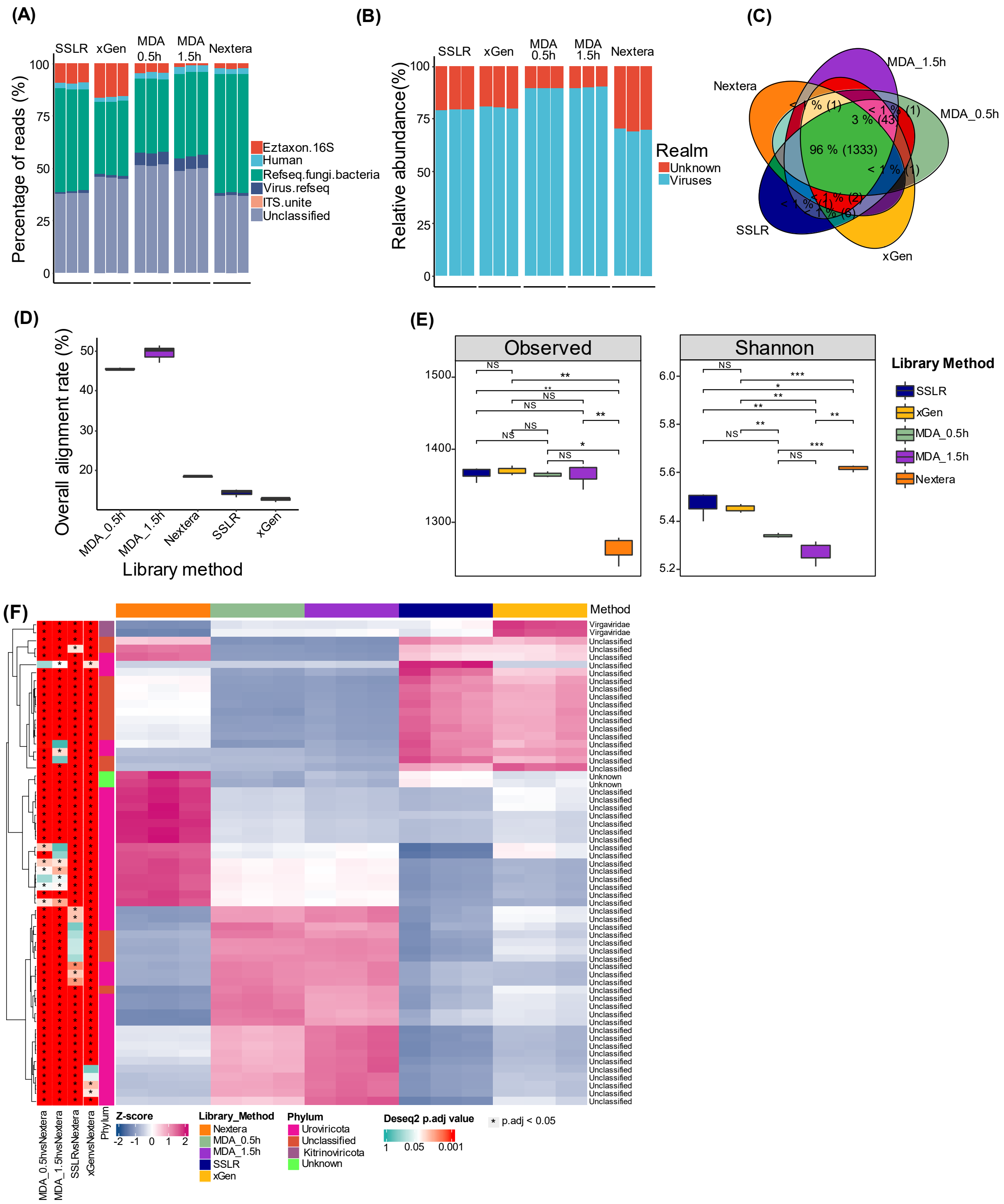

Fig. S8 | (A) Distribution of sequencing reads into the different taxonomic categories as viral, human, bacterial, fungi, and unknown origin. To check the presence of non-viral DNA sequences, 50,000 random forward reads were evaluated according to their match to a range of viral, bacterial, and human reference genome and protein databases. No reads (in 50,000 reads) matched the 18S rRNA gene sequences in all the samples. (B) The ratio of viruses and unknown parts of fecal samples prepared with different methods. (C) The Venn diagram indicates the presence of vOTUs in the different library preparation methods. (D) The overall alignment rate (%) of high-quality contigs that mapped to viral ref database. (E) The effect of different library preparation methods on the viral overall-alpha diversity with the measurement of Observed and Shannon index. NS indicates not significant, one asterisk indicates a

significant difference at $p < 0.05$ (t.test), two asterisk indicates a significant difference at $p < 0.01$ (t.test), three asterisk indicates a very significant difference at $p < 0.001$ (t.test). (F) Differential abundance analysis by comparing the tested library preparation methods on the distribution of viruses at the family level (Nextera XT library method was used as reference). Hierarchical clustering was performed to group family (rows) and library preparation methods (columns) based on similarities in differential abundance signatures and was visualized via heatmap.

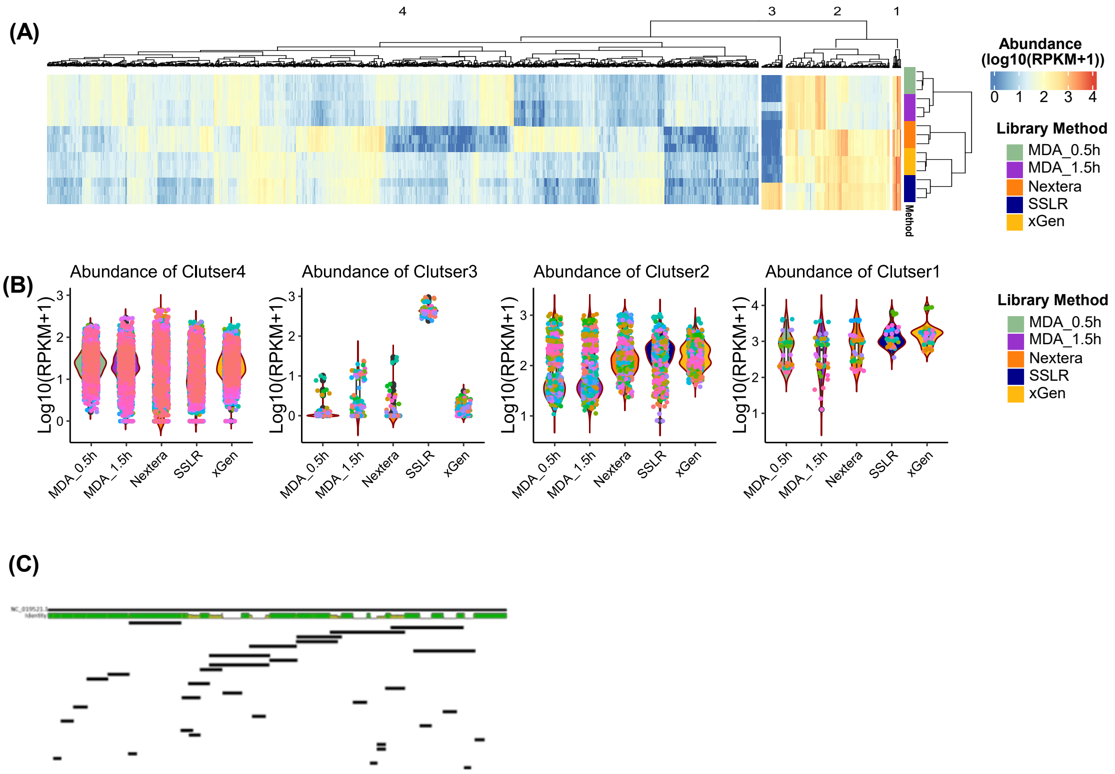

Fig. S9 | (A) The heatmap shows the abundances (log10(RPKM+1)) of FV contigs, and the virome contigs were divided into 4 clusters. (B) The abundances of the 4 divided clusters. (C) 34 of high abundance (RPKM) viral contigs that were highly enriched in the SSLR method that are not present in the rest of the methods. Contig was piped through VirSorter 2 and DRAMv for viral identification and annotation. Multiple hits matched to Sphingomonas phage PAU (NC_019521.1).

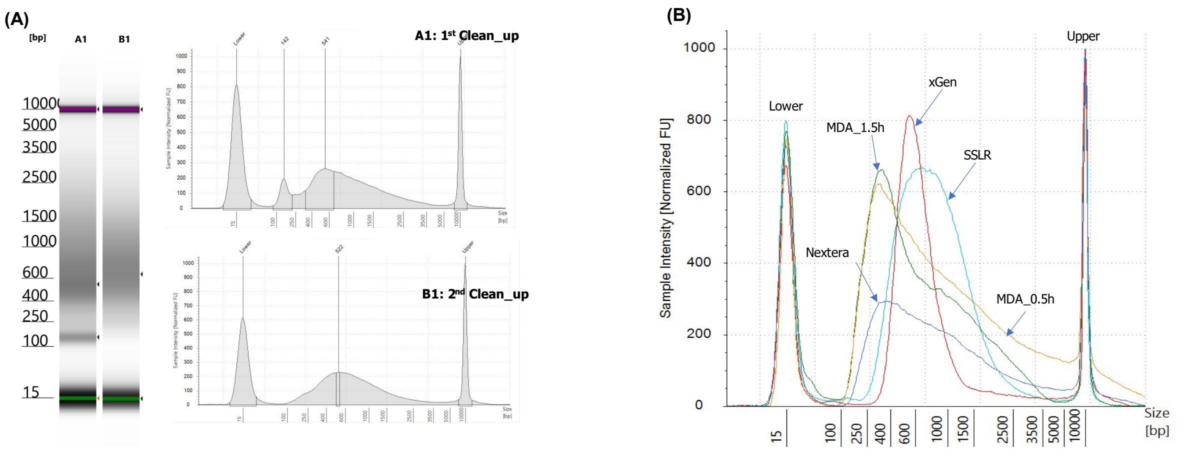

Fig. S10 | Library size assessment by Agilent TapeStation 4200. (A) The extra clean-up step can remove the dimers that the first-round bead clean-up cannot remove. The fecal virome library was prepared by the SSLR and cleaned up by 0.8x AMPure XP beads. (B) Different library methods result in different library size distributions. In general, both MDA and Nextera XT tend to have shorter library sizes compared to single-stranded methods (SSLR and xGen).
